# Increased connectivity of hiPSC-derived neural networks in multiphase granular hydrogel scaffolds

**DOI:** 10.1016/j.bioactmat.2021.07.008

**Published:** 2021-07-15

**Authors:** Chia-Chen Hsu, Julian H. George, Sharlayne Waller, Cyril Besnard, David A Nagel, Eric J Hill, Michael D. Coleman, Alexander M. Korsunsky, Zhanfeng Cui, Hua Ye

**Affiliations:** a Institute of Biomedical Engineering, Department of Engineering Science, University of Oxford, OX3 7DQ, UK; b MBLEM, Department of Engineering Science, University of Oxford, Parks Road, Oxford, OX1 3PJ, UK; c School of Biosciences, College of Health and Life Sciences, Aston University, Birmingham, B4 7ET, UK; d Translational Medicine Research Group, Aston Medical School, College of Health and Life Sciences, Aston University, Birmingham, B4 7ET, UK

**Keywords:** Microgel, Hydrogel, Hyaluronan, iPSC, Neural tissue engineering, 3D printing

## Abstract

To reflect human development, it is critical to create a substrate that can support long-term cell survival, differentiation, and maturation. Hydrogels are promising materials for 3D cultures. However, a bulk structure consisting of dense polymer networks often leads to suboptimal microenvironments that impedes nutrient exchange and cell-to-cell interaction. Herein, granular hydrogel-based scaffolds were used to support 3D human induced pluripotent stem cell (hiPSC)-derived neural networks. A custom designed 3D printed toolset was developed to extrude hyaluronic acid hydrogel through a porous nylon fabric to generate hydrogel granules. Cells and hydrogel granules were combined using a weaker secondary gelation step, forming self-supporting cell laden scaffolds. At three and seven days, granular scaffolds supported higher cell viability compared to bulk hydrogels, whereas granular scaffolds supported more neurite bearing cells and longer neurite extensions (65.52 ± 11.59 μm) after seven days compared to bulk hydrogels (22.90 ± 4.70 μm). Long-term (three-month) cultures of clinically relevant hiPSC-derived neural cells in granular hydrogels supported well established neuronal and astrocytic colonies and a high level of neurite extension both inside and beyond the scaffold. This approach is significant as it provides a simple, rapid and efficient way to achieve a tissue-relevant granular structure within hydrogel cultures.

## Highlights

• In longer-term culture, granular hydrogels supported the development of neural culture with extensive neurite outgrowth.
• Granular hydrogels supported significantly higher cell viability and neurite outgrowth over 7 days of culture.
• A simple 3D printed extrusion and mixing toolset is used to generate and homogenously seed cells into granular hydrogels.
• The toolset designs are provided for 3D printing, enabling use and customization of the techniques described.

## Graphical abstract

**Figure:**
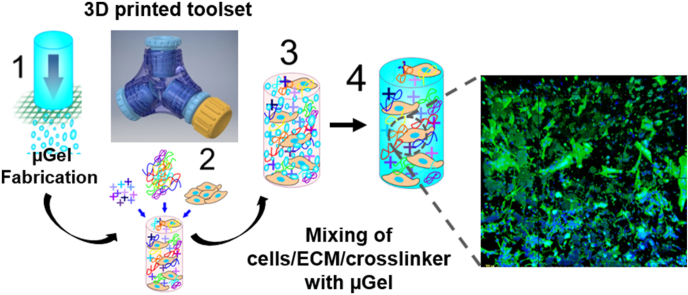

## 1 Introduction

The development of *in vitro* neural tissue analogs is of considerable interest in the field of biomedical engineering, particularly for disease modeling and drug screening applications. Widely used conventional two-dimensional (2D) cultures differ from brain tissue in many ways, spatially limiting cell-to-cell and cell-to-extracellular matrix (ECM) interactions and lacking cues and structures that support and pattern cells, growth factors, nutrients, and waste exchange *in vivo* [1]. Three-dimensional (3D) model systems can be used to more closely replicate *in vivo* human neural development. More recently, sponges and electrospun fibrous scaffolds used as culture substrates have been shown to have beneficial effects on neural survival, proliferation, differentiation, and neurite outgrowth [[2], [3], [4], [5], [6], [7], [8], [9], [10]]. Organoid techniques that allow for the growth and patterning of human pluripotent stem cells (PSCs) into multiple cell types has the importance of cell-to-cell interactions in 3D human neural tissue development *in vitro* [11,12]. Despite tremendous efforts to create 3D neural cultures *in vitro*, development of reproducible models that support longer-term neural network development remains challenging. During cultures, issues such as slow exchange of nutrients, oxygen and waste into and out of cell agglomerations and bulk materials, as well as the inability to dynamically modulate cell‐to-cell and cell-to-matrix interactions still need to be overcome [13].

Hydrogels are highly hydrated 3D crosslinked polymer networks. As tissue culture scaffolds, they are extremely customizable and their low material density and high levels of hydration permit cellular accessibility, useful in microscopy and other types of investigation [14]. Through modification of material stiffness, porosity and the addition of biological factors, it is feasible to generate highly tailored niche microenvironments for *in vitro* cell culture and *in vivo* transplantation. More recently, hydrogels have become one of the most well-established and commonly used materials for tissue engineering applications. Bulk hydrogels (i.e. hydrogels crosslinked in-place into a continuous volume) have been used to augment the delivery of biological factors [[15], [16], [17], [18]], acting as highly tailorable support matrices, bridging damaged tissues, and to serving as cell carriers that support cell viability whilst preventing cell loss in cell therapies [[19], [20], [21]]. However, crosslinking hydrogel around cells in suspension entraps the cells in a nanoscale mesh that can limit outgrowth, connectivity, tissue development and basic cellular physiological processes [22]. One way to overcome these challenges is to modify bulk hydrogel by breaking it into a granular structure and placing cells at the discontinuity boundaries between granules. Furthermore, the assembled hydrogel granular matrix can be used as a modular biomaterial implant analogous with complex native tissue structures that have interconnected micropore or mesopore systems [[23], [24], [25]]. Cell cultures within granule-like hydrogel structures have previously been investigated *in* *vitro* and *in* *vivo*. In one study, rat hippocampal neurons cultured on chitosan hydrogel microparticles *in* *vitro* were found to form 3D interconnected and electrophysiologically active neural networks [26]. In this study, cells were plated onto the surface of microbeads. To ensure homogeneous cell distribution, the beads in cell suspension required multiple repositioning and remixing during plating over a 3-4-h incubation period. In another study *in* *vivo*, a macroporous hydrogel composite scaffold formed using polyethylene glycol (PEG) microparticles was transplanted into a mouse spinal cord injury model [23]. The resulting tissue growth in the multi-phase scaffold led to functional recovery, with robust axon elongation, myelination and decreased glial scarring. However, the fabrication of the type of scaffold used required multiple additional steps including the removal of excess photoinitiator, molding, and ethanol sterilization prior to transplant. Use of granular hydrogel scaffolds with sterile toolsets can significantly simplify the complexity of scaffold fabrication and seeding, making these types of study more accessible.

A significant advantage of granular hydrogel scaffolds is that the granules can be fluidized and undergo shear-thinning upon compression [27], facilitating transport and localized placement whilst also shielding cells from shear stress during injection [28]. This is of particular interest for *in* *vivo* applications. For example, hyaluronic acid (HA)-based microgels injected into a stroke cavity model and annealed *in situ* supported tissue growth with reduced inflammation, increased vascularization, promoting neural progenitor proliferation and migration into the stroke site [29]. Fragmented chitosan microgels injected into a rat spinal cord injury model were similarly found to enhance spinal tissue repair [30]. Granular hydrogel systems have also been used for cell delivery, drug delivery and other tissue engineering applications [[31], [32], [33], [34], [35]], including bone and cartilage regeneration [[36], [37], [38], [39]], as well as the growth of skin [24,40] and heart tissue [[41], [42], [43]].

Many different techniques have been used to fabricate hydrogel microparticles, including the use of batch emulsions, microfluidic emulsions, template lithography, electrohydrodynamic spraying, and mechanical fragmentation [44,45]. Of these techniques, microfluidic emulsions, lithography and electrohydrodynamic spraying provide the highest degree of control over individual particle shape and size; however, production tends to be low-throughput and scaling-up can be challenging and costly [45]. In contrast, batch emulsions or mechanical fragmentation can be used to rapidly produce large amounts of granular hydrogel, albeit with increased variability in particle size and shape [[45], [46], [47], [48]]. Importantly, mechanical fragmentation does not require the subsequent removal of extraneous solutions, such as emulsion chemicals, greatly simplifying the use of this technique in biologically sensitive applications.

Mechanical fragmentation is advantageous in its speed and simplicity [45], enabling rapid scale-up. Various methods have been used for mechanical fragmentation, including blending hydrogels in a homogenizer or a consumer-grade blender [30,49], as well as mechanically forcing hydrogels through a steel mesh [47]. Fragmentation of chitosan hydrogel in a homogenizer produced granules sized between 20 μm and 150 μm, with size dependent on rotation speed and blending time [30]. Gelatin hydrogels blended with a consumer-grade blender decreased in size with increased blending time, with a blending time of 120 seconds resulting in microparticles with a mean Feret diameter of 55.3 ± 2 μm [49]. In this study forced extrusion through a porous mesh was used to produce results in gel fragments with a controlled size distribution related to mesh pore size. Previously, polycarboxybetaine hydrogels fragmented using a steel mesh have been found to produce microgel fragments 15–30 μm in diameters [47].

Granular hydrogel scaffolds facilitate homogenous cell seeding, better control over local cell-to-cell density and provide biologically relevant structure within culture scaffolds; however, the adoption of granular scaffolds presents unique challenges. Issues such as hydrogel loss during fabrication, granule size variability and granule dispersion in culture medium can cause complications to experimental setup and limit outcomes. Furthermore, understanding how granular scaffolds can be used to facilitate cell seeding, modulate cell viability and support longer-term cultures has not been well established. In this study these issues are explored through the culture of human induced pluripotent stem cell (hiPSC)-derived cortical neurons in granular hyaluronan hydrogel scaffolds. A custom designed 3D printed toolset was used to fabricate granular hydrogel scaffolds. The toolset designs are made available to increase the accessibility and customizability of the techniques used. Due to the toolset's sterilizability, we do not require complicated sterilization methods, and the enclosed design of our toolset avoids waste of materials during hydrogel extrusion and seeding. Hyluronan was chosen as a base substrate as it is a major constituent of the proteoglycan rich environment of developing neural tissue [50]. Insufficient nutrient and metabolic waste exchange and inhibition of cell-cell interaction can occur within bulk hydrogels [51], and use of multiphase granular scaffolds may overcome these issues, presenting a promising approach for long-term *in vitro* 3D neural culture, supporting complex neural network development.

## 2 Materials and Methods

### 2.1 Cell culture

hiPSCs, line 010S-1, was derived at the Highfield Unit, Warneford Hospital, Oxford from a skin biopsy of an 18-year-old female healthy subject. hiPSCs were maintained on Matrigel™ (Corning, UK)-coated culture plates using Essential 8 media (Thermo Fisher Scientific, UK) and mTeSR™1 media (STEMCELL Technologies, UK) and were passaged using 0.5 mM EDTA (pH 8.0; Thermo Fisher Scientific) in phosphate buffer solution (PBS) when they reached appropriate confluency. Neural differentiation was based on previously published protocols with some modifications [5,[52], [53], [54]]. hiPSC cultures were used for neural conversion when reached confluence. Neural Basal Medium was prepared by mixing [Neurobasal Medium (Thermo Fisher Scientific), 2 v/v % B27 Supplement (Invitrogen), 1 v/v % MEM Non-Essential Amino Acids (Thermo Fisher Scientific), 1 v/v % penicillin/streptomycin (Invitrogen), 1 v/v % GlutaMAX (Invitrogen)] and [Advanced DMEM/F-12 medium (Thermo Fisher Scientific), 1 v/v % N-2 supplement (Invitrogen, UK), 0.2 v/v % B27 Supplement (Invitrogen), 1 v/v % penicillin/streptomycin (Invitrogen), 1 v/v % GlutaMAX (Invitrogen)] at 1:1 ratio. The cells were differentiated via dual SMAD signaling inhibition [55], using Neural Basal Medium supplemented with SB431542 (10 μM; Calbiochem, UK) and InSolution™ AMPK Inhibitor, Compound C (2 μM; Calbiochem, UK)] for 7–10 d. Differentiated cells were passaged on laminin from Engelbreth-Holm-Swarm murine sarcoma basement membrane (10 μg/ml; Sigma-Aldrich, UK)-coated plates in the Neural Basal Medium. After 3–5 d, the cells proliferated and formed neural rosette structures. The culture medium was changed into Neural Basal Medium supplemented with 20 ng/ml Recombinant Human FGF-basic (bFGF; PeproTech). These derived neural progenitor cells (NPCs) were passaged every 5–7 d using Accutase (STEMCELL Technologies) on laminin-coated plates for the first few passages and on Matrigel-coated plates for later passages. Differentiation of the NPCs into neurons was performed using Neural Basal Medium supplemented with 10 ng/ml glial cell-derived neurotrophic factor (GDNF; PeproTech) and 10 ng/ml brain-derived neurotrophic factor (BDNF; PeproTech).

Another hiPSC‐derived NPC line used in this study was AXOL13 cells, purchased from Axol Bioscience, UK. NPCs (50,000 cells/cm^2^) were plated onto laminin-coated plates in Neural Plating Medium (Axol Bioscience, UK) for 24 h. Cultures were maintained in Neural Maintenance-XF Medium (Axol Bioscience) and neuronal differentiation was performed using Neural Differentiation-XF Medium (Axol Bioscience) in accordance with the manufacturer's protocols. The line 010S-1 hiPSCs were used in cell viability and neurite outgrowth studies and both line 010S-1 and AXOL13 cells were used in long-term culture studies.

### 2.2 Fabrication and assembly of 3D printed tools

**Fig. 1:**
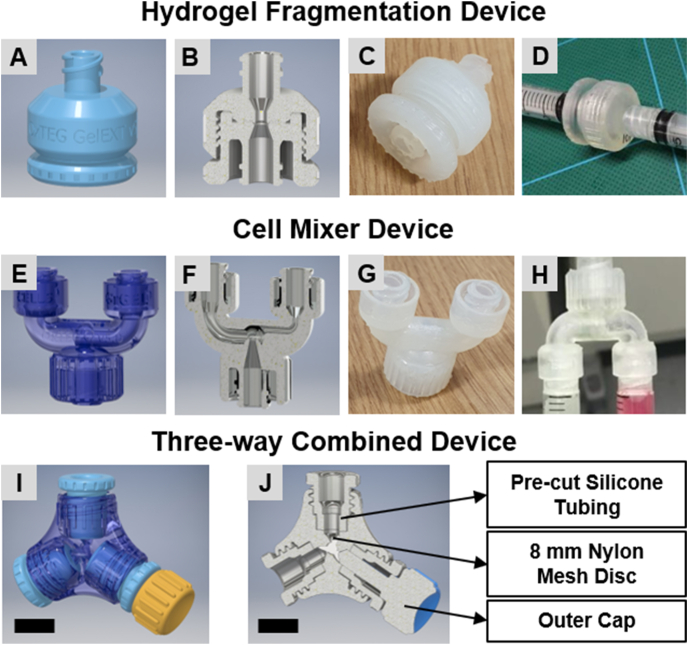
Designed 3D printed toolsets for granular hydrogel composite fabrication. (A–D) Hydrogel fragmentation device, (E–H) Cell mixer device, and (I–J) Three-way combined device, which merges the hydrogel fragmentation device and the cell mixer device (scale bars = 1 mm).

To improve the ease and repeatability of hydrogel granule fabrication and the homogeneity of cell and granular gel mixing, three types of work devices were designed and fabricated, including a hydrogel fragmentation device, a cell mixer device, and a three-way combined device, where the hydrogel fragmentation device and the cell mixer device were merged into a single device (Fig. 1).

Working prototypes of the 3D printed toolset were designed using AutoCAD Inventor software (Autodesk, USA) and realized with high-resolution 3D printing on a Form2 printer (FormLabs, USA). Inventor software was used to generate stereolithography (STL) files that were processed using PreForm software (FormLabs, USA) prior to printing. The STL files and FORM files of the 3D Printed Toolkit have been made available on the Open Science Framework (https://doi.org/10.17605/OSF.IO/6XK3Q).

The Form2 printer uses Durable Resin v2 (Express Group Ltd, UK) that contain photo-initiators and a layer-by-layer laser-based photo-polymerization process to fabricate parts that have a minimum XY feature size of 200 μm and a minimum layer height of 25 μm. Following printing, parts were washed in 70 v/v % ethanol (2 × 10 min) to remove non-polymerized resin and post cured under UV light. For the gel fragmentation device, polydimethylsiloxane (PDMS, Sylgard 184, Corning, UK) was cured for 4 h at 60 °C onto the surface of the part that contacts an 8 mm nylon mesh disc. This PDMS layer provided a compliant surface that was needed to form a seal around the nylon mesh inside the device. To test the devices, 2 w/v % HyStem® hydrogel (fabricated as described in Section 2.3) was extruded through the assembled device between two 2.5 ml luer-lock syringes (Fig. 1A–D), and the hydrogel fragments were mixed with an equal volume of media, together with 20 v/v % of the primary crosslinker concentration by pressing the two solutions through the mixing device using 2.5 ml lure-lock syringes (Fig. 1E–H). The mixed solution was collected into a second 2.5 ml syringe and gel discs were cast from the resulting mixture. The gel fragmentation device and the mixer device were further merged into a three-way combined device (Fig. 1I and J). During the assembly process, an 8 mm nylon mesh disc was placed on one of the three channels for gel fragmentation and three pieces of 3 mm (length) x 4 mm (inner diameter) x 8 mm (outer diameter) clear silicone tubing were then placed inside the device for all three channels before fastening with their outer caps to avoid leakage. To test gel fragmentation using the three-way combined device, 2 w/v % HyStem® hydrogel was extruded through the channel installed with the nylon disc between two 1 ml luer-slip concentric tip syringes while the spare channel was blocked and sealed with a custom designed plug. To test cell mixing using the three-way combined device, the hydrogel fragments were mixed with an equal volume of media (with the secondary crosslinker of 20 v/v % of the primary crosslinker concentration) by pressing the two solutions back and forth three times between two 1 ml luer-slip concentric tip syringes while the gel fragmentation channel was blocked and sealed with the plug.

### 2.3 Bulk and granular hydrogel fabrication

The hydrogel used in this study was fabricated using a commercially available kit, HyStem® (Sigma-Aldrich), consisting of HyStem® (recombinant derived thiolated hyaluronan precursors, Mw 158 kDa) and Extralink-1 (polyethylene glycol diacrylate (PEGDA), Mw 3.4 kDa), previously characterized [[56], [57], [58]]. 2 w/v % hydrogels were used and the hydrogels were fabricated following a modified version of the manufacturer's recommended protocols. Briefly, 500 μl of degassed water was added to the HyStem® component and allowed to dissolve on a roller mixer at room temperature for 30 min, making a 2 w/v % HyStem® solution. The Extralink-1 crosslinker supplied with the kit was reconstituted as 2 w/v % concentration with 250 μl degassed water. Degassed water was used in precursor solutions to mitigate the effects of oxidative crosslinking caused by oxygen exposure of thiol groups with disulfide links [56,57].

**Fig. 2:**
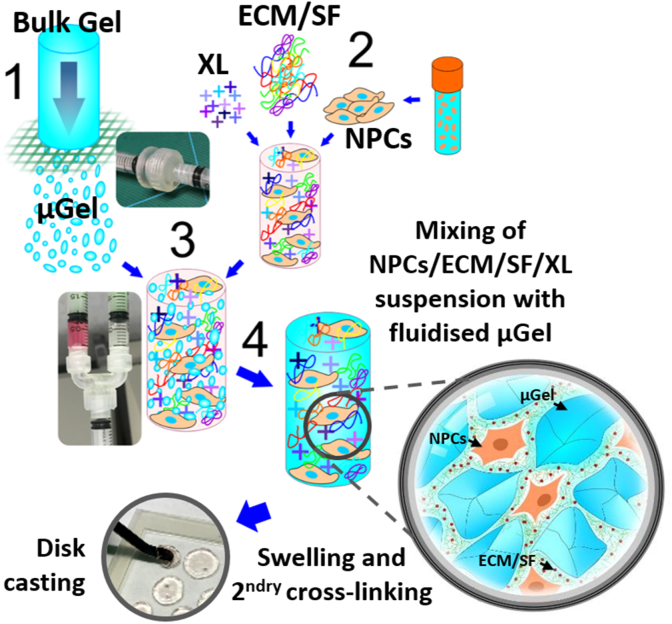
Schematic of multiphase hydrogel fabrication, including hydrogel fragmentation, cell mixing process, secondary crosslinking, and gel disc casting (XL: crosslinker; ECM: extracellular matrix; SF: soluble factor; NPCs: neural progenitor cells; μgel: microgel).

For bulk hydrogels, 1:1 (v:v) ratio of PBS/medium and 150 μl of the 2 w/v % Extralink-1 were added to the 500 μl 2 w/v % HyStem®. The system was gelled in a 1 ml syringe at 37 °C for 30 min to form a final volume of 1 w/v % bulk hydrogel. For granular hydrogels, 125 μl of the 2 w/v % Extralink-1 was mixed with 500 μl 2 w/v % HyStem® and the system was pre-gelled in a 1 ml syringe at 37 °C for 2.5 h. Once gelled, gel fragments were formed by extruding the hydrogel through the gel fragmentation device or the gel fragment channel of the three-way combined device with an 8 mm nylon mesh disc and the gel fragments were collected using a syringe. 1:1 (v:v) ratio of PBS/medium and the secondary Extralink-1 crosslinker with various concentrations was added to the gel fragments and mixed using the cell mixing device or the cell mixing channel of the three-way combined device. The mixed system was collected in a syringe and gelled at 37 °C for 30 min to form a final volume of 1 w/v % granular hydrogel and extruded into a cell culture dish. Schematic diagram of the fabrication process of the granular hydrogel composite is shown in Fig. 2. Alternatively, without the addition of a secondary crosslinker, use of wells or chambers made by agarose assembled with porous membranes could assist to hold the mix of cells and hydrogel granules together. To maintain the structural integrity of the granular hydrogel composites, laminin was refreshed each time cell culture media was replaced.

### 2.4 Characterization and analysis of hydrogel granule size

Fragment size was characterized using optical microscopy following hydrogel fragmentation through nylon mesh of different pore sizes. Discs were punched from cell sieves and filters using a biopsy punch (8 mm diameter). Cell sieves with nylon mesh pore diameters of 40 μm and 70 μm were sourced from Fisher Scientific (UK) and nylon mesh filter discs with 5 μm and 10 μm pore diameters were obtained from Millipore (UK). To form the hydrogel, 2 w/v % HyStem® hydrogel were fabricated as previously described in Section 2.3. Once gelled, hydrogel fragments were formed by extruding the hydrogel through the nylon mesh discs with various pore sizes and repeated three times to generate fine gel fragments. The process of mechanical fragmentation was achieved in <5 min and these fragments were collected in a sterile Petri dish and 100 μl of the gel fragments were mixed with 1 ml of PBS. This solution was then run across a 9 cm petri-dish held at a 45° angle and excessive PBS was removed such that individual gel fragments could be visualized using a brightfield imaging system with differential interference contrast (DIC). This method measures the size of swollen hydrogel granules, which better represents the size and morphology seen during cell culture in media. Images were taken within 10 min of placing the gel fragments onto the dish to avoid dehydration of the gel. ImageJ software was used for image analysis to calculate the average diameters of the gel fragments.

### 2.5 Rheological characterization

Micro-rheological characterization of bulk and granular hydrogel gelation was performed using Diffusing Wave Spectroscopy system (DWS RheoLab; LS instruments, CH). With the use of this technique, speckle patterns resulting from the interaction of a 650 nm laser and 600 nm diameter tracer particles (1 w/v %) entrapped in the hydrogel were analyzed to evaluate how gel network interaction modulated the tracer particle's thermal motion. For bulk hydrogels, various concentrations of HyStem® precursors (0.5, 1.0, 1.5, or 2.0 w/v %) were reconstituted with corresponding amounts of degassed water and fabricated following the manufacturer's recommended protocol. Briefly, a 10 w/v % solution of 600 nm polystyrene tracer particles (Sigma-Aldrich, UK) were added to the gel precursors together with 1x Extralink-1 crosslinking solution. The mixture was added to 2 mm thick glass cuvettes (LS Instruments, CH), sealed with a lid and wrapped in parafilm to prevent dehydration, and then placed into the micro-rheology system. Using a custom script created using the RheoLab 6.2 software (LS Instruments, CH), recordings were taken at 37 °C for 30 cycles (each cycle was constituted of 30 s multi-tau mode measurement, followed by 30 s echo mode measurement) with no time delay between each cycle. To examine micro-rheological properties of granular hydrogels, 2.0 w/v % hydrogel fragments with 10 w/v % 600 nm polystyrene tracer particles were formed following the procedures described in Section 2.3 using a 40 μm pore diameter nylon mesh. A 10 w/v % solution of 600 nm polystyrene tracer particles in PBS was mixed with the same amount of the hydrogel fragments, together with the secondary crosslinker at 10, 20, 30, or 50 v/v % of the primary crosslinker concentration. The mixture was then added to a 2 mm thick glass cuvette and the cuvette was sealed as described above to prevent dehydration. Measurements were recorded at 37 °C for 30 cycles (each cycle was constituted of 30 s multi-tau mode measurement, followed by 30 s echo mode measurement) with no time delay between each cycle. For data analysis, a custom python script (created using PyCharm community edition software, JetBrains, UK) was used to collate the data from the generated text files (one per time point) and the storage modulus G′ was plotted for equivalent oscillatory mode at frequency of 10 and 100 Hz for granular hydrogels and bulk hydrogels, respectively.

### 2.6 Cell encapsulation in bulk and granular hydrogels and gel casting

For cell encapsulation in granular hydrogels, 2 w/v % HyStem® hydrogel fragments were fabricated as shown in Section 2.3 using a pre-sterilized three-way combined device after autoclave. 5 × 10^4^ cells/cm^2^ hiPSC-derived NPCs were seeded on TCPs as the 2D control for baseline cell viability and performance. To achieve similar cell density, ~10^7^ cells/ml was chosen by scaling up the cell numbers seeded on 2D surfaces into 3D culture (see Table S1. Equivalent cell seeding densities for 2D controls and 3D cubic hydrogel models). 2 × 10^7^ cells/ml of hiPSC-derived NPCs were first resuspended in F20 Medium supplemented with 200 μg/ml laminin and 50 ng/ml BDNF. 400 μl of hydrogel fragments were then mixed with an equal volume of the cell suspension, together with the secondary crosslinker at 20 v/v % of the primary crosslinker concentration by pressing the hydrogel fragments and cell solution back and forth three times between two 1 ml luer-slip concentric tip syringes while the gel fragment channel of the three-way combined device was blocked and sealed with the custom designed plug. The final granular hydrogel composites with ~10^7^ cells/ml hiPSC-derived NPCs were incubated and gelled at 37 °C for 30 min after adding the secondary crosslinker. For bulk hydrogels, 400 μl of 2 w/v % precursor HyStem® component was mixed with an equal volume of the cell suspension, together with the same amount of total Extralink-1 crosslinker added into the granular hydrogel composites using a 1 ml syringe. The system was gelled in the syringe at 37 °C for 30 min after adding the crosslinker to form the final bulk hydrogel of ~10^7^ cells/ml hiPSC-derived NPCs. After hydrogel formation, the bulk and granular hydrogels were cast into discs using two slices of glass slides with space thickness of 500 μm in between. The hydrogel discs of 500 μm thickness were gently translocated into glass-bottom dishes (Greiner Bio-One Ltd, UK), topped up with Neural Basal Medium supplemented with 10 ng/ml BDNF and 10 ng/ml GDNF for neuronal differentiation, and maintained by half media exchange every 3 days.

### 2.7 Immunostaining and fluorescence microscopy

Cell-hydrogel constructs were fixed in 4.0 v/v % paraformaldehyde (Sigma-Aldrich) in PBS for 30 min at room temperature, blocked and permeabilized in 0.2 v/v % Triton X-100 (Sigma-Aldrich) and 5 v/v % bovine serum albumin (Sigma-Aldrich) in PBS for 1–2 h at room temperature. Each step described above was followed by three washes with PBS for 5 min. The constructs were then incubated for overnight at 4 °C in 0.2 v/v % Triton X-100 and 0.2 v/v % bovine serum albumin (Sigma-Aldrich) in PBS with primary antibodies, Nestin (1:500; Millipore), GFAP (1:1000; Sigma-Aldrich), Neurofilament (1:1000; Abcam, UK), and βIII-tubulin (1:1000; Sigma-Aldrich) (Table S2). After a minimum of 3 washes with PBS for 1 h on the shaker at low speed, the constructs were incubated for overnight at 4 °C in 0.2 v/v % Triton X-100 and 0.2 v/v % bovine serum albumin in PBS with NucBlue Live ReadyProbes™ Reagent (Invitrogen) or Hoechst 33342 staining solution (Thermo Fisher Scientific), and Alexa Fluor secondary antibodies (Thermo Fisher Scientific), followed with 3 washes with PBS for 1 h on the shaker at low speed. The stained samples were stored at 4 °C and the images were acquired with a Nikon Eclipse Ti-E inverted fluorescence microscope (Nikon Instruments Inc., UK).

### 2.8 Cell viability assay

Human iPSC-derived NPCs were encapsulated within bulk and granular hydrogels, as well as seeded onto control tissue culture polystyrene (TCP) plates in Neural Basal Medium supplemented with 10 ng/ml BDNF and 10 ng/ml GDNF. The cell seeding densities were ~10^7^ cells/ml and 5 × 10^4^/cm^2^ for the hydrogels and TCP controls, respectively. Cell viability was evaluated on Day 1, Day 3, and Day 7 using a live cell stain, Calcein AM (Abcam), and DRAQ5™ (Abcam), which is a cell permeable far-red fluorescent DNA dye staining cell nuclei. Images for cell viability assay were acquired with a Nikon TiE C2+ confocal microscope (Nikon Instruments Inc., UK) by sequential scanning. The thickness of the acquired hydrogel sections was about 500 μm and z stacks of typically 79 × 6.5 μm slices were imaged. The cell viability was determined by the percentage of the number of viable cells to the total number of cell nuclei analyzed using Imaris Image Analysis Software (Oxford Instruments plc, UK). A filter was applied to remove the background noise based on the particle size (>524 μm^3^), which correlates to the soma diameter of the NPCs of 10 μm.

### 2.9 Neurite outgrowth of induced pluripotent stem cell-derived neurons

For morphological analysis of neurons, confocal images were acquired with a Nikon TiE C2+ confocal microscope by sequential scanning. To observe neurite outgrowth, a smaller step size along the z-axis was taken for a higher z-axis spatial resolution. The acquired hydrogel sections were about 300 μm thick with z stacks of typically 95 × 3.2 μm slices. 3D imaging software Avizo version 2020.1 and earlier versions (Thermo Fisher Scientific) were used for neurite outgrowth analysis. Voxel size of 0.62 (x) × 0.62 (y) × 3.2 (z) μm was defined by the image acquisition parameters. Neurite tracing was performed using the Filament Editor workroom of the Avizo XFiber extension module, which manually extracts individual neuronal traces in 3D by determining nodes along the z-axis. Each of the traced neurites was labelled as a graph and the neurite length was extracted from each graph. Neurite outgrowth was evaluated by statistical data analysis on the average neurite length and the number of neurite bearing cells. Following the identification of neurites, a spatial graph containing all the extracted neurites overlapped with the raw data were present in 3D using the volume rendering and spatial graph view modules.

### 2.10 Statistical analysis

For statistical analysis, all experiments were conducted three times with three technical replicates in each cell experiment. For cell viability, the results were from three independent experiments with three technical replicates and at least three different fields were analyzed per sample. For neurite outgrowth, the results were from three independent experiments with three technical replicates and two different fields were analyzed per sample. Ten neurites per imaged field were traced if possible (for some fields, less than 10 neurites were identified) and a total of ~180 neurites per group were analyzed at each time point. One-way ANOVA with post hoc Tukey's test and two-sample *t*-test were used throughout the study and specified in figure legends. A p-value <0.05 was considered statistically significant and all results represent means ± SEM unless specified otherwise. (In the diagrams, * represents p < 0.05, ** represents p ≤ 0.01, and *** represents p ≤ 0.001.)

## 3 Results and discussion

### 3.1 Formation and microstructure of granular HA hydrogels

**Fig. 3:**
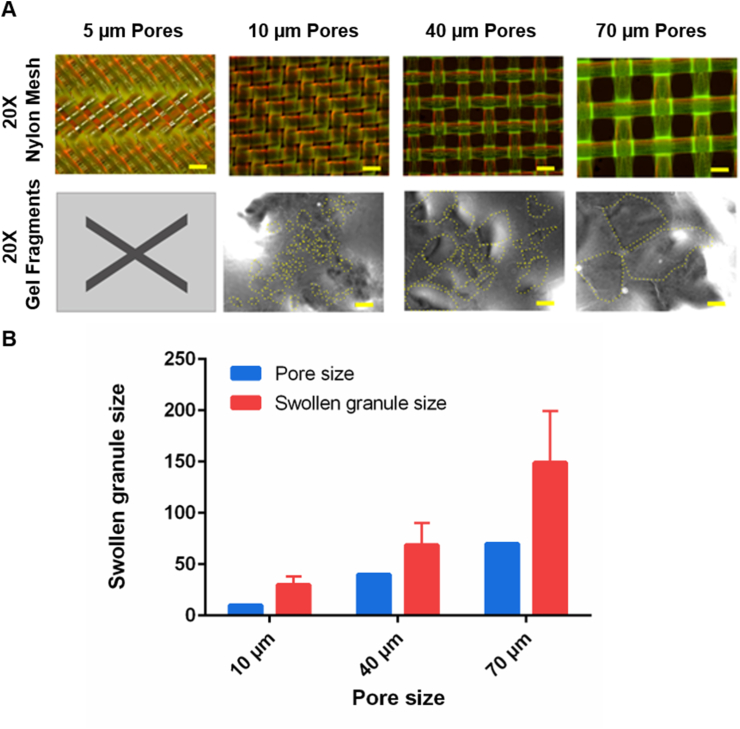
Microstructures of extruded hydrogel granules. (A) Hydrogel granules fabricated with nylon mesh weaves of 10, 40, and 70 μm pore sizes. The fabricated gel granules are highlighted with a yellow border having been fluidized in PBS and imaged using transmitted white light illumination with differential interference contrast (DIC) (scale bars = 50 μm). (B) Gel granule size is proportional to the pore diameter of nylon meshes with uniform pore diameters which are commercially available (The results represent means ± SD. N = 26, 18, and 6 for 10, 40, and 70 μm pore sizes, respectively. * represents p < 0.05 and ** represents p ≤ 0.01.).

To fabricate the granular scaffolds used in this study, a 3D printed extrusion and mixing tool was developed with low chamber volume, reducing hydrogel loss during fabrication (see https://doi.org/10.17605/OSF.IO/6XK3Q). Thiol-functionalized hyaluronan hydrogel (HyStem™, Sigma-Aldrich, UK) was crosslinked and extruded through a fine sterile woven nylon fabric placed into the custom extrusion chamber between the two syringes. During optimization, different concentrations of hydrogel were evaluated. It was found that 4 w/v % hydrogels were too viscous to extrude, whilst 1 w/v % hydrogels resulted in swollen granules that were weak and not suitable for longer-term culture. 2 w/v % hydrogel granules swell to ~1 w/v % hydrogel when mixed 1:1 with cells suspended in culture medium. The size and shape of the 2 w/v % hydrogel granules were characterized using optical microscopy (Fig. 3 and Figure S1).

Extrusion of hydrogel through the nylon mesh resulted in irregularly shaped granules (Fig. 3A). Following extrusion, these granules expanded to more than twice the diameter of the extrusion pores (granular size ~ 2x nylon mesh pore size). Specifically, extrusion through fabric with pore widths of 70 μm, 40 μm and 10 μm resulted in average granule diameters of 149 ± 50 μm, 69 ± 21 μm and 30 ± 8 μm, respectively (Fig. 3B). The force required to extrude hydrogel though the nylon fabric increased with decreasing pore diameter, such that it became impossible to manually extrude 2 w/v % hydrogel through 5 μm diameter pores.

The ability to easily control hydrogel granule size enables their use to be tailored to experimental requirements and to fit the scale of relevant biological features. For example, mature human neurons typically have soma diameters of 10–50 μm, whilst axons and dendrites have diameters of 0.2–3 μm [59]. Whilst it may be desirable to match the size of these biological features directly, a compromise must be made between scaling down to match cell and dendrite size and scaling up granule size to reduce the overall combined surface area of the granules. This combined surface area (effectively the size of the absorption space between granules) acts to dilute the coverage of both secondary crosslinkers and biological factors added during cell-mixing. In this study gel granules extruded through 40 μm pore diameters and having diameters of 69 ± 21 μm were chosen as a suitable compromise between these two considerations.

### 3.2 Rheological analyses of bulk HA hydrogels

**Fig. 4:**
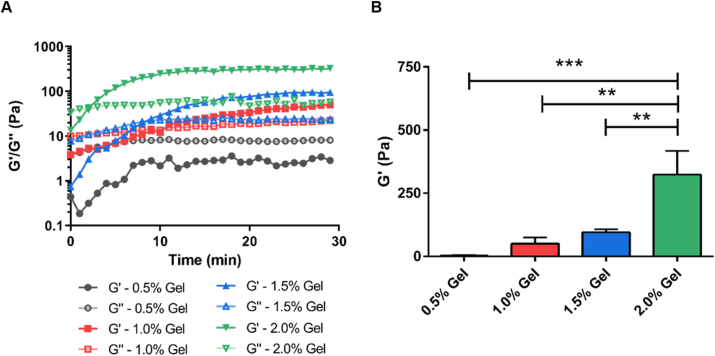
Rheological analyses of bulk HA hydrogels. (A) Gelation kinetics (G′ and G″) of bulk HA hydrogels made of various concentrations (0.5, 1.0, 1.5, and 2.0 w/v %) of HyStem® precursors. (B) Storage moduli (G′) of bulk HA hydrogels with various concentrations of HyStem® precursors after 30 min gelation measured by DWS RheoLab. (One-way ANOVA with post hoc Tukey's test was used. The results represent means ± SEM. N ≥ 3. ** represents p ≤ 0.01 and *** represents p ≤ 0.001.)

Hydrogel scaffold mechanical properties have been found to play a significant role in determining cellular responses. Micro-rheology (DWS RheoLab, LS instruments, CH) was used to determine the mechanical properties of PEGDA-crosslinked thiol-modified hyaluronan hydrogel, using multi-tau and echo measurement modes (Fig. 4 and Figure S2). The storage modulus (G′) and the loss modulus (G″) of the hydrogels were low at the beginning of gelation with G′ lower than G″. As gelation proceeded, the value of G′ gradually increased and became higher than that of G″, indicating the formation of the crosslinked hydrogel network. The gelation point, where G′ and G″ intersect, marks the hydrogel's liquid-solid transition boundary [60]. For all groups, except the 0.5 w/v % mix that did not form a hydrogel, gelation occurred within 10 min and hydrogels reached their ultimate storage modulus within 30 min. The shortest gelation time (~2 min) was observed for the 2.0 w/v % hydrogel. The highest G′ value was measured for the most concentrated hydrogel formulation (324 ± 93 Pa for 2.0 w/v %), whereas significantly lower G′ values were recorded for lower hyaluronan hydrogel concentrations (95 ± 7 Pa for 1.5 w/v %, 50 ± 12 Pa for 1 w/v % and 3 ± 1 Pa for 0.5 w/v %). This decrease in G′ with gel precursor concentration follows directly from the increase in relative hydration and the decrease in effective crosslinking density and physical entanglement between hydrogel polymer chains. A significant variability in mechanical properties was observed between different batches of the commercially sourced hydrogels tested (Figure S1). Despite this, the storage moduli of each group were found to be significantly different based on One-way ANOVA analysis (Fig. 4B).

### 3.3 Rheological analyses of granular HA hydrogels

Unbound hydrogel granules disperse when placed into culture medium and need to be contained within a chamber. To overcome this, a secondary weak crosslinking step can be used to loosely bind granules together into a scaffold that is self-supporting in culture medium. The secondary crosslinker can be added at the time of cell mixing, together with cells and other biological factors. To determine the effect of this additional secondary crosslinker on swelling dynamics and scaffold mechanical properties, micro-rheology was performed on tracer particles encapsulated within hydrogel granules that were mixed (1:1) with the secondary crosslinker solution. Subsequent granule swelling, enmeshing, and secondary crosslinking leads to a reduction in tracer particle free motion, enabling the overall increase in system storage modulus to be measured. It was not possible to directly compare the rheological properties of the bulk and granular hydrogels as the location and compartmentalization of the tracer particles differs between the two systems.

**Fig. 5:**
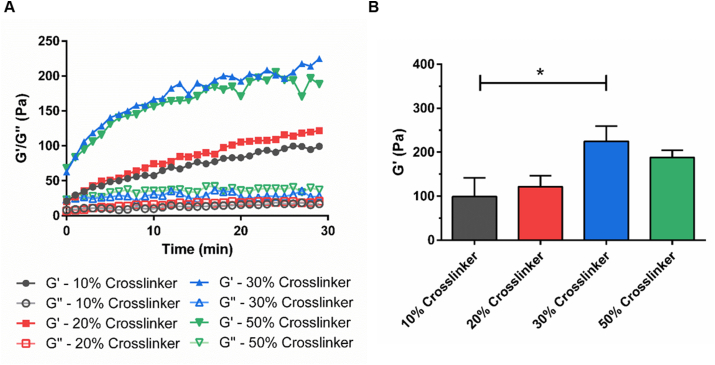
Swelling of granular hydrogel composites and effects of secondary crosslinking on rheology. (A) Gelation kinetics (G′ and G″) of granular HA hydrogel composites made of 2.0 w/v % hydrogel granules and secondary crosslinkers at 10, 20, 30, or 50 w/v % of the primary crosslinker concentration. (B) Storage moduli (G′) of granular HA hydrogels with various concentrations of secondary crosslinker after 30 min gelation. (One-way ANOVA with post hoc Tukey's test was used. The results represent means ± SD. N = 3. * represents p < 0.05.)

Storage modulus (G′) was found to increase throughout the 30-min investigation while the loss modulus (G″) of all granular hydrogels maintained low with G′ higher than G′′ (Fig. 5 and Figure S3). Following 30 min of gelation, granular hydrogel mixed with solutions of secondary crosslinker at 10 v/v %, 20 v/v %, 30 v/v % and 50 v/v % (relative to the primary crosslinker concentration) acquired G′ values of 99 ± 43 Pa, 121 ± 25 Pa, 225 ± 60 Pa and 188 ± 28 Pa, respectively. Granular hydrogel of 30 v/v % secondarily crosslinker resulted in systems with significantly higher G′ values compared to that of 10 v/v % secondarily crosslinker (Fig. 5B). It was found that cells cultured within the stiffer granular hydrogel composites (with secondary crosslinkers at 30 v/v % and 50 v/v % of the primary crosslinker concentration) formed tight cell clusters (Figure S4). Granular hydrogels bound with the secondary crosslinker at 10 v/v % of the primary crosslinker concentration were mechanically weak and broke apart in culture. Granular scaffolds with a secondary crosslinker at 20 v/v % of the primary crosslinker concentration were chosen for further study, as they promoted low levels of cell clustering whilst also remaining intact throughout the 28-day culture period.

The mechanical properties of a culture substrate are known to modify cell behaviors, and this is especially true for neural cells. For this reason, hydrogel polymer choice, chain length, density, and crosslinking regimes are often tailored to match tissue properties. For example, thiol-modified HA crosslinked with PEGDA can be tailored to match a range of storage moduli ranging between 11 Pa and 3500 Pa by varying macromer and crosslinker concentrations [61]. NPC self-renewal and neural differentiation has been shown to occur more readily in softer matrices (E ~ 100–1000 Pa) [[62], [63], [64]], and in particularly, softer hydrogels (~100–500 Pa) have been previously found to promote neuronal differentiation, while stiffer hydrogels (~1000–10,000 Pa) have been found to enhance glial differentiation [65,66]. Neuronal maturity has been shown to be enhanced on soft substrates [67]. Furthermore, for NPCs encapsulated in 3D alginate hydrogels, both proliferation and differentiation of the encapsulated NPCs were found to be significantly increased when the elastic modulus of the alginate hydrogel decreased [68].

In this study, the storage modulus of both the bulk and granular hyaluronan hydrogels was found to fall within the range known to promote NPC proliferation and differentiation [[64], [65], [66],^68^]. However, for bulk hydrogel, a compromise exists between choosing a hydrogel that will support cells and remain stable throughout extended culture periods, whilst also being soft enough to permit neurite extension. In contrast, for granular hydrogels, cells are seeded into the space between hydrogel granules, such that the cell perceives a micro-environment that is both stiff at the granule boundary for attachment and, at the same time, weak between granules to support neurite extension and cell migration. This granular boundary intersection microenvironment forms a biologically relevant niche that can be tailored to better match the organization of the chosen tissue type.

### 3.4 Cell viability in granular hydrogel composites

**Fig. 6:**
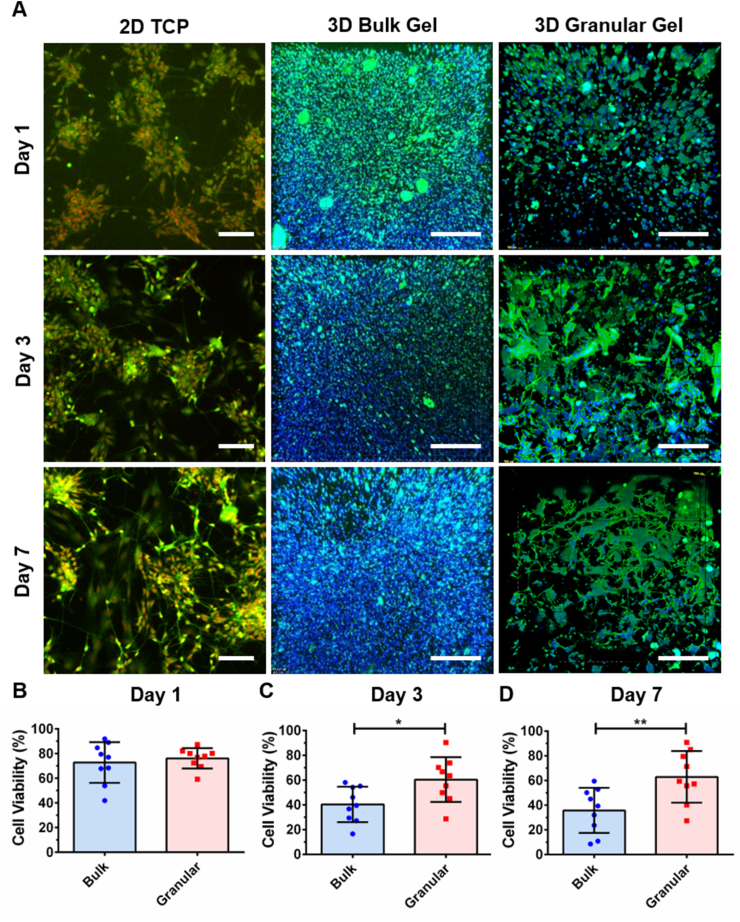
Cell viability of hiPSC-derived NPCs in bulk and granular hydrogel constructs. (A) Fluorescent images of hiPSC-derived NPCs on tissue culture polystyrene (TCP) as well as bulk and granular hydrogel constructs at Day 1, Day 3, and Day 7 (Calcein AM, green; DRAQ5™, red/blue; scale bars = 100 μm). Cell viability as the percentage of live cells among total cell nuclei in bulk and granular hydrogel systems was examined on (B) Day 1, (C) Day 3, and (D) Day 7. (Two-sample *t*-test was used. The results represent means ± SEM. N = 3; n = 9. * represents p < 0.05 and ** represents p ≤ 0.01.).

Biocompatibility of granular hydrogel composites were evaluated using clinically relevant hiPSC-derived neuronal populations. Unlike primary cultures from animal models or immortalized cell lines commonly used in *in vitro* modelling systems, the use of clinically relevant cell populations can facilitate the translation of basic research into clinical applicability. Confluent hiPSCs were directed toward neuroectoderm via dual SMAD signaling inhibition [55]. The derived NPCs proliferated and formed characteristic neural rosette structures, which appear during early hiPSC neural development [69]. After dissociating and incorporating hiPSC-derived NPCs into bulk hydrogels and granular hydrogel composites, it was found that the granular hydrogel scaffolds supported higher levels of cell viability in comparison to the bulk hydrogel scaffolds. Cell viability was compared at 1, 3 and 7 days. No significant difference in cell viability was observed on the first day of culture (bulk cell viability: 73 ± 6%; granular cell viability: 76 ± 3%) (Fig. 6). Previous studies have reported that shear-thinning behaviors of hydrogels can protect cells from shear stresses applied during cell-hydrogel mixing and syringe injection [27,28,45]. While extruding cells in non-crosslinked solutions, the shear thinning behaviors in bulk hydrogels may transfer shear forces to encapsulated cells, thus affecting cell viability. In our experimental set up, encapsulated cells were surrounded by crosslinked bulk hydrogel or stable hydrogel granular composites and cell viability during mixing and following syringe extrusion remained high. However, mixing and extruding with different pressures and speeds was not investigated. After three days in culture, cell viability in granular hydrogels was found to be significantly higher than for cells encapsulated in bulk hydrogel (bulk cell viability: 40 ± 5%; granular cell viability: 60 ± 6%) and after seven days in culture, cell viability in the granular hydrogel scaffolds remained significantly higher (Bulk: 36 ± 6%; Granular: 63 ± 7%). Culture on 2D surfaces (not being comparable to 3D culture results) [70] demonstrated overall high levels of cell viability. Unlike immortalized neural cell lines that are known to be extremely tolerant [5,71], hiPSC-derived NPCs are inherently sensitive to 3D culture conditions and their response is likely to be more representative of overall scaffold biocompatibility.

Maintaining higher levels of cell viability in hydrogel culture is complex and depends on multiple factors. For example, the viability of rat NPCs cultured in Hyaluronan hydrogel was found to decrease from ~80% to ~40% over 24 h [72]. Decreasing cell viability has also been seen in mouse and human NPC cultures encapsulated in self-assembled peptide, Matrigel, and collagen hydrogels [21,73]. Often the rate of cell death increases over time as cells proliferate and form tight clusters. This level of cell death demonstrates a fundamental failure of these culture environments, indicating that cells are either stressed or not provided with the necessary cues and factors needed for survival. In our study, there was a significant decrease in cell viability in bulk hydrogels at Day 3 (p ≤ 0.001) and Day 7 (p ≤ 0.001) compared to Day 1. However, there was no significant difference in cell viability observed in granular hydrogel composites at Day 3 (p = 0.134) and Day 7 (p = 0.233) compared to Day 1, demonstrating that granular hydrogel composites pose minimal impacts to cell viability in short term cell culture.

Cells mixed into granular gels can be spaced homogenously during seeding, cells in the granular hydrogel composite were found to distribute homogenously in all three dimensions (Figure S5), suggesting that nutrients throughout the scaffold were consumed more homogeneously during the early culture period. The granular architecture was also thought to provide a more permissive environment for cell migration as there are interspaces between hydrogel granules and the additional secondary crosslinker may create interconnected micropore or mesopore systems (Figure S6-S7), where an average pore size of 1378 ± 157 (mean ± SEM) μm^2^ was found based on SEM image analysis (Figure S8; n = 3, 1353 pores analyzed). Furthermore, whilst untethered factors can quickly diffuse out of hydrogel matrices and become lost from the culture [73], it is possible that regions of enriched ECM deposited by cells between granules entrapped factors, slowing their release, consumption, and degradation. Cell viability in granular hydrogel composites was similar to that observed in HA hydrogel networks chemically tethered with BDNF [72]. Cell viability was used to evaluate the biocompatibility of the granular hydrogel composites in this study. It would be of interest to also determine how other cell responses, such as cell proliferation and metabolic activity, are modulated by granular scaffold architecture in future studies.

### 3.5 Neurite outgrowth and extension in granular hydrogel composites

**Fig. 7:**
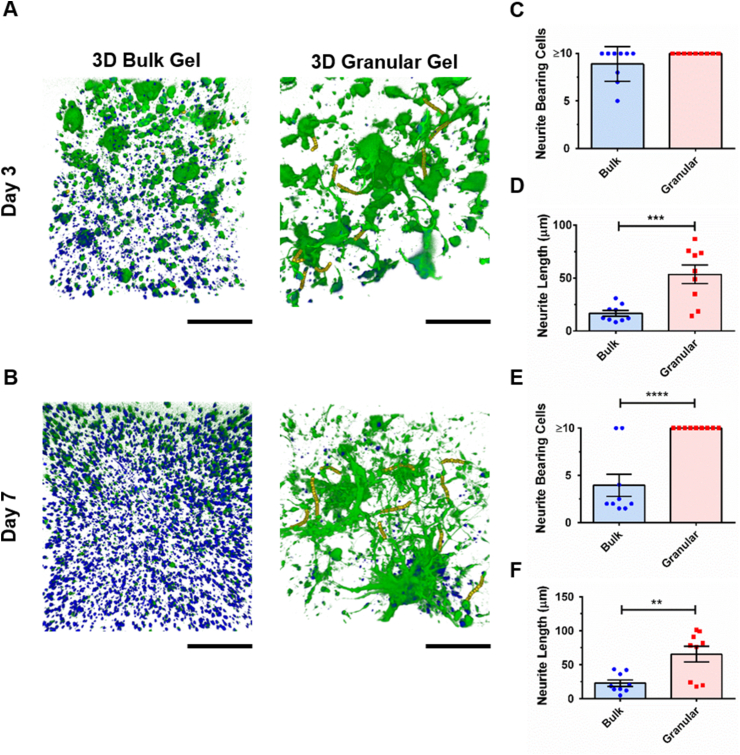
Neurite outgrowth and neurite network formation in bulk and granular hydrogel constructs. 3D reconstruction of confocal images of cells in bulk and granular hydrogels at (A) Day 3 and (B) Day 7 (Calcein AM, green; DRAQ5™, blue; neurite tracing, yellow; scale bars = 200 μm). Number of neurite bearing cells at (C) Day 3 and (E) Day 7 as well as the average neurite length at (D) Day 3 and (F) Day 7 were examined as indications of neurite outgrowth and neural network formation. (Two-sample *t*-test was used. The results represent means ± SEM. N = 3; n = 9. ** represents p ≤ 0.01, *** represents p ≤ 0.001, **** represents p ≤ 0.0001.).

Granular HA hydrogels were found to promote and support neurite extension. Confocal microscopy was used to image neurite bearing cells and analyze neurite length in bulk and granular hydrogels. Measurement of neurite length was performed directly on the 3D reconstructed confocal models, rather than by tracing on compressed 2D projection images [19,74,75]. Cell morphology in the two groups was found to be significantly different, with cells taking on very different morphologies over time (Fig. 7, Movie S1–S4). Analysis was performed by counting neurites on up to 10 randomly selected cells in each 3D image volume. After three days in culture, both hydrogel scaffolds were seen to support similar numbers of neurite bearing cells (Bulk: 8.89 ± 0.61; Granular: 10.0 ± 0.00; p = 0.088). However, the length of neurites extending from cells in the granular gel far exceeded neurite length in bulk hydrogels (Bulk average neurite length: 16.69 ± 2.63 μm; Granular average neurite length: 53.38 ± 8.72 μm) (Fig. 7D). After seven days of culture, the number of neurite bearing cells was significantly decreased in bulk hydrogel (Bulk: 3.94 ± 1.17; Granular: 10.0 ± 0.00; p < 0.0001). Neurite length in bulk hydrogel, whilst longer than on the third day of culture, was on average three times shorter than neurite length in granular hydrogels (Bulk average neurite length: 22.90 ± 4.70 μm; Granular average neurite length: 65.52 ± 11.59 μm). In comparison, neurons cultured for seven days in methacrylated HA hydrogels were found to have average neurite lengths of ~14 μm in stiffer bulk hydrogel (1.41 ± 0.27 kPa) and ~49 μm in softer bulk hydrogels (0.51 ± 0.20 kPa) [76].

Supplementary video related to this article can be found at https://doi.org/10.1016/j.bioactmat.2021.07.008

The following is the supplementary data related to this article:

Video 2 Video

Video 3 Video

Video 4 Video

Video 5 Video

Whilst bulk hydrogels have been widely used as drug delivery vehicles and cell carriers in tissue engineering studies, cell morphology and process extension are often not analyzed or reported in the early stages of culture [[77], [78], [79], [80], [81]]. Neurite branching and extension are significant morphological changes during neuronal differentiation and maturation [82], and neurite outgrowth in scaffolds is a good indicator of the scaffold's ability to support the essential process of cell attachment and extension. Neurite extension is impeded by the cell's surrounding microenvironment that may lack sufficient adhesive ligands to support cell attachment and migration. Laminin is one of the main components in developing neural tissues and is known to enhance cell adhesion, growth, and differentiation [83,84]. To support cell attachment, laminin was incorporated into both the bulk and granular hydrogels. Neurite extension can also become impeded by the surrounding matrix forming an impenetrable densely crosslinked polymeric shell around the neuron [[78], [79], [80]]. For granular scaffolds, the space between hydrogel granules may act in a similar way to interconnected void spaces in porous solid scaffolds, allowing for the rapid diffusion of nutrients and cell signaling factors [27,85], and providing pathways to support cell migration and neurite extension [29]. In comparison to scaffolds fabricated by other granular hydrogel fabrication methods (e.g. using microfluidic devices or batch emulsion), mechanically fragmented granular hydrogel scaffolds have been shown to be more porous, while void space fraction is similar [48]. This increased porosity may further facilitate cellular interaction. For microgels fabricated using microfluidic water-in-oil droplet segmentation, it was found that the median pore size of the microporous network increased with microgel diameter [86]. In a different study, mechanically fragmented granular hydrogels with an average granule diameter of ~100 μm were found to have a void space of ~8% and a median pore size of ~100 μm^2^ [48]. Cells respond differently to biophysical and biochemical environments. For example, human mesenchymal stem cells were found to increase migration speed on concave spherical structures in comparison to flat surfaces and convex spherical structures [87]. This was thought to result from differences in cytoskeletal forces generated on each surface type, leading to nuclear deformation and promoting varying degrees of osteogenic differentiation.

Whilst the effects of hydrogel granule size are unknown, granule size may indirectly modulate protein distribution within a granular scaffold. Smaller granules have a greater relative outer surface area than larger granules for the same mass of hydrogel. If larger proteins collect on granular boundaries during seeding, a scaffold composed of larger granules would accrue a relatively greater amount of protein per surface area from a fixed concentration solution than a scaffold composed of smaller granules. This variation in protein concentration could in turn modulate cell behavior. While a wide range of topographical length scales and geometries have been developed to study the outgrowth and functions of neural stem cells [88], the effects of hydrogel granular size on regulating cell proliferation, differentiation, neurite extension and migration have not been well studied. The custom designed toolset is well suited to facilitate this type of investigation.

### 3.6 Long-term culture of hiPSC-derived NPCs in granular hydrogel composites

**Fig. 8:**
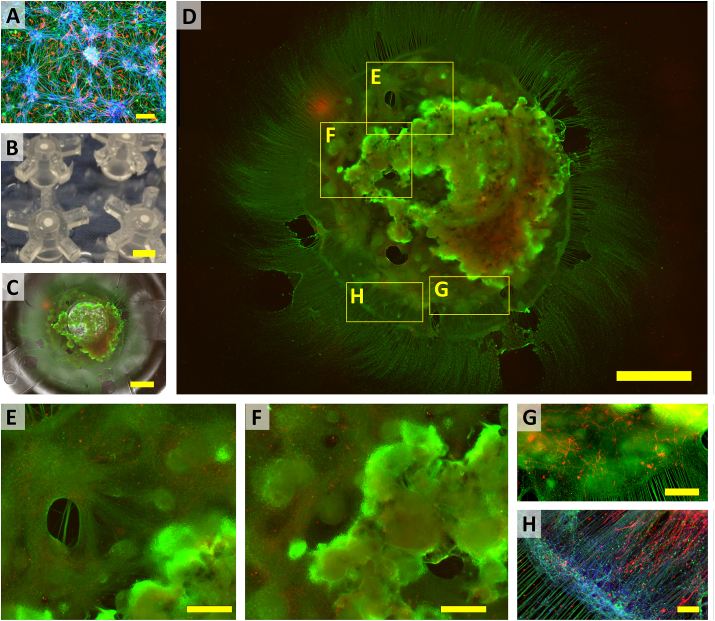
Long-term culture (3 months) of AXOL13 NPCs in granular hydrogel composites. (A) hiPSC-derived neurons grew to form connected neural networks on tissue culture polystyrene (TCP) (scale bar = 100 μm). (B) Image of inverted PDMS 12-well plate microchambers assembled with porous membranes (white circles; scale bar = 10 mm). (C) Composite epifluorescence and brightfield image of granular microgel neural culture in PDMS chamber (scale bar = 5 mm). (D) Enlarged epifluorescent image of granular microgel neural culture (scale bar = 5 mm). (E–H) Close-up of sections marked in (D), showing (E) Cellular clusters with extensive neurite growth inside the granular microgel scaffold (scale bar = 100 μm), (F) Large densely packed cellular clusters (scale bar = 100 μm), (G) Astrocyte growth between the neural clusters in the periphery of the culture (scale bar = 100 μm), and (H) Well aligned neurite and astrocyte extensions in the periphery of the culture (scale bar = 100 μm). Immunostaining reveals neurons (Tuj1, Green), astrocytes (GFAP, Red), and axons (Neurofilament, Blue).

To investigate the longer-term culture potential, stem cell-derived neural cells were seeded into granular hydrogel scaffolds and cultured for one or three months, then assessed by immunostaining with neuronal (anti-Tuj1) and astrocytic (anti-GFAP) markers (Fig. 8 and Figure S9-S10). Axons were stained with an anti-neurofilament antibody to observe axon outgrowth of the differentiated neurons. When grown in conventional monolayer culture, NPCs differentiated into both astrocytes and neurons that form connected clusters spreading across the culture plate (Fig. 8A). To perform longer-term culture in hydrogels, PDMS crowns were fabricated to fit into the wells of a 12-well plate and hold the 3D cultures in a static position below a central well that was used to add culture medium (Fig. 8B). The cultures remained in place under the central well and grew neurites that extended out onto the plate (Fig. 8C). Imaging also revealed that hiPSC-derived NPCs proliferated and differentiated into large densely packed cellular clusters connected by large bundles of neurites. These cell clusters were seen to contain populations of both neurons (Tuj1+ cells) and astrocytes (GFAP + cells) (Fig. 8D–G). The neuronal coverage was higher than astrocytic coverage after 1 month and 3 months culture and both neuronal and astrocytic coverage increased over time (Figure S10A). During neural development in vertebrates, neurogenesis precedes gliogenesis [89]. Astrocytes are known for supporting neurons for synaptogenesis, neuronal regeneration, and neurite outgrowth and guidance [89,90]. Astrocytes were also observed between the neuronal clusters and were well aligned with the neurites in the peripheral region of the culture (Fig. 8H). The neuronal clusters were surrounded by extensive neurite growth both inside the granular microgel scaffold and extending well aligned neurite projections beyond the hydrogel scaffold. The extended neurites or axons were found to cross over long distances and the mean length of neurites/neurite bundles was increased over time from 1.2 mm at 1 Month to 4.0 mm at 3 Month (Figure S10B-D), demonstrating the capability of the granular hydrogel system to support cell culture, neural differentiation, and neurite elongation over long periods. Electrophysiology of human iPSC derived neurons in hydrogel culture has been demonstrated elsewhere [91]. Whilst no attempt was made to record electrophysiological activity from the long-term cultures, the presence of bundles of neurites between the neural clusters suggests the formation of neural networks.

Whilst culture of neural cells in biomaterials is well established, relatively few studies investigate longer-term neural growth in the culture matrix. Bulk collagen and self-assembling peptide hydrogels have been used for culturing adult mouse neural stem cells (NSCs) for up to 5 months [73]. The study reported non-preferential differentiation of NSCs into neurons, astrocytes, and oligodendrocytes after three-month in culture and neuronal cells were found to appear earlier than glial phenotypes. However, neurite extension and network formation in long-term culture were not reported. Matrigel, a basement membrane extract, has been widely used in neural organoid cultures. Differentiated PSCs embedded in Matrigel can be cultured for 3–6 months, undergoing self-organization to form organoids in-part resembling tissue structures found *in vivo* [92]. The resulting neural organoids contain many different cell types, including radial glial cells, neuroepithelial progenitors, neuronal lineage cells and astrocytes [93,94], with cell diversity increasing over time [93]. In this study, use of stably crosslinked hydrogels was chosen to support longer-term culture, and the presence of neurons and astrocytes was examined *in situ* using confocal microscopy. Scaffold degradation can also be used to release and isolate cells for characterization. This approach requires the use of materials that can be degraded without damaging cells. For example, thermoresponsive hydrogels have been used for cell encapsulation and harvesting [95,96]. However, these cultures tend to become unstable in longer-term studies. The selection of suitably stable degradable materials that support longer-term culture will be the focus of future studies.

Although considerable effort has been made to develop different types of 3D hydrogel substrate for long-term cell culture, most 3D culture studies still rely on the use of biologically derived ECM constructs, such as Matrigel [13]. While these native ECM hydrogels present a highly biocompatible and permissive scaffold, high batch-to-batch variability and the xeno- and tumorigenic origins of Matrigel are often cited as drawbacks [51]. Chemically defined synthetic polypeptide hydrogels with the flexibility that can be tailored for tissue engineering applications have also been used for neural culture [51,73]. However, unmodified ECM extract and polypeptide hydrogels degrade relatively quickly in culture and often require the supplementation of additional ECM proteins as a dissolved component to prolong culture stability [51]. Granular hydrogel systems, based on chemically defined hydrogels, remain stable throughout extended culture periods and represent a promising approach to cell culture for long-term studies.

The custom designed 3D printed toolset presented in this study can fabricate multiphase hydrogels contain ECM, cells and soluble factors. To enhance cell performances, granular gels can be functionalized to increase the retention of soluble bioactive factors. HyStem®-HP hydrogel contains similar components as the HyStem® hydrogel in combination with a thiol-modified heparin, which forms an ionic bond with proteins, thus facilitating the slow release of the encapsulated bioactive factors and ensuring its release in close proximity to the cells [97]. Other hydrogel polymers which can immobilize soluble factors via covalent immobilization (e.g. carbodiimide coupling immobilization [98]), physical immobilization (e.g. biotin–streptavidin interactions [99]) and ECM-inspired immobilization (e.g. heparin or adhesive protein-based binding methods [100,101]) can also be adapted as the base material for granular hydrogel composites to support long-term soluble factor release [102].

## 4 Conclusion

In this study, granular hydrogel scaffolds were optimized for the culture of human iPSC-derived cortical neurons and cells were seeded into the laminin-rich weak gel layer formed between swollen hydrogel granules. The optimized granular hydrogel scaffolds supported significantly higher cell viability and neurite outgrowth over 3 and 7 days in culture. Furthermore, the granular hydrogel construct was able to support longer-term neural culture over three months, demonstrating that the scaffolds can support the development of both neuron and astrocyte colonies with extensive neurite outgrowth and complex neurite network.

A 3D printed manual extrusion and mixing toolset was created to simplify hydrogel granule generation and seeding with minimal hydrogel loss. Granular hydrogel scaffolds are highly customizable and can be readily adapted for use in modern patterning techniques, whilst their relative simplicity also makes this approach suited to applications in a clinical setting. The feasibility of using the designed 3D printed toolset for granular hydrogel fabrication should be extensible to other materials and other cell types with tunable materials and particle sizes. The multiphase cell encapsulating hydrogel system developed in this study can be valuable for *in vitro* cell culture models for disease modeling and drug screening as well as for the potential treatment of neurological diseases.

## Supporting Information

Supporting Information is provided for further results, including size distribution of extruded hydrogel granules in Figure S1, rheological analyses (G′ and G″) of bulk and granular HA hydrogels in Figure S2 and S3, SH-SY5Y cell encapsulation in granular hydrogel composites in Figure S4, homogenous cell distribution in granular hydrogels in Figure S5, characterization of internal structure of bulk and granular hydrogels in Figure S6, SEM images and image analysis of bulk hydrogels and granular hydrogel composites in Figure S7 and Figure S8, long-term culture of hiPSC (line 010S-1)-derived NPCs in a granular hydrogel composite in Figure S9, analysis of neural differentiation and neurite extension in long term neural culture in granular hydrogel composites in Figure S10, and neurite outgrowth and extension in bulk and granular hydrogel constructs in Movie S1–S4. Materials and Methods with equivalent cell seeding densities for 2D controls and 3D cubic hydrogel models in Table S1 and a list of antibodies used for immunostaining in Table S2 as well as details on the SH-SY5Y neuron culture was included.

## Notes

The authors declare no competing financial interest. The research materials supporting this publication can be accessed by contacting Prof Hua Ye.

## CRediT authorship contribution statement

**Chia-Chen Hsu:** Conceptualization, Writing – original draft, Investigation, Methodology, Visualization, Formal analysis. **Julian H. George:** Conceptualization, Methodology, Writing – review & editing, Investigation, Visualization. **Sharlayne Waller:** Writing – review & editing, Methodology, Investigation. **Cyril Besnard:** Writing – review & editing, Formal analysis. **David A Nagel:** Writing – review & editing, Methodology. **Eric J Hill:** Writing – review & editing, Methodology. **Michael D. Coleman:** Writing – review & editing, Funding acquisition. **Alexander M. Korsunsky:** Writing – review & editing, Formal analysis. **Zhanfeng Cui:** Conceptualization, Writing – review & editing, Supervision, Funding acquisition. **Hua Ye:** Conceptualization, Methodology, Writing – review & editing, Supervision, Project administration, Funding acquisition.
