# Supplementary material for: Increased connectivity of hiPSC-derived neural networks in multiphase granular hydrogel scaffolds
Source: Bioact Mater. 2021 Jul 15;9:358–72. doi: 10.1016/j.bioactmat.2021.07.008 (PMC8586009; doi:10.1016/j.bioactmat.2021.07.008)
Supplement: Multimedia component 1 [file mmc1.pdf]

Supporting Information

Increased Connectivity of hiPSC-derived Neural Networks in Multiphase Granular Hydrogel Scaffolds

Chia-Chen Hsu[a], Julian H George[a], Sharlayne Waller[a], Cyril Besnard[b], David Nagel[c, d], Eric Hill[c], Michael D Coleman[c], Alexander M. Korsunsky[b], Zhanfeng Cui [a], Hua Ye[a*]*

[a]Institute of Biomedical Engineering, Department of Engineering Science, University of Oxford, OX3 7DQ, United Kingdom

[b]MBLEM, Department of Engineering Science, University of Oxford, Parks Road, Oxford OX1 3PJ, United Kingdom

[c]School of Biosciences, College of Health and Life Sciences, Aston University, Birmingham B4 7ET, United Kingdom

[d]Translational Medicine Research Group, Aston Medical School, College of Health and Life Sciences, Aston University, Birmingham B4 7ET, United Kingdom

[*]Corresponding author. Institute of Biomedical Engineering, Department of Engineering Science, University of Oxford, OX3 7DQ, United Kingdom. E-mail address: hua.ye@eng.ox.ac.uk (H. Ye).

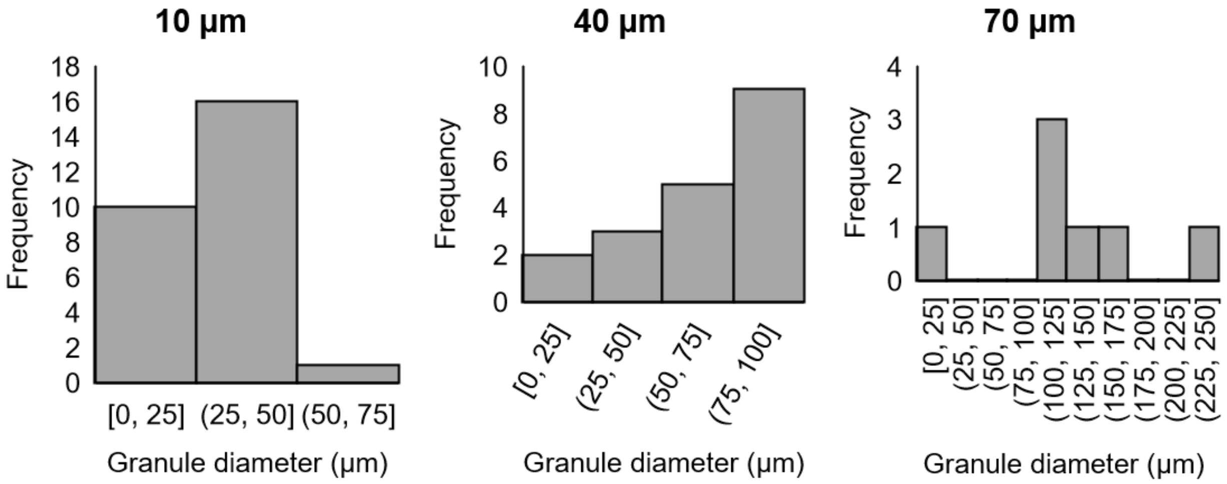

Figure S1. Size distribution of extruded hydrogel granules fabricated with nylon mesh weaves of 10, 40, and 70 μm pore sizes. (The results represent means ± SD. N = 26, 18, and 6 for 10, 40, and 70 μm pore sizes, respectively.)

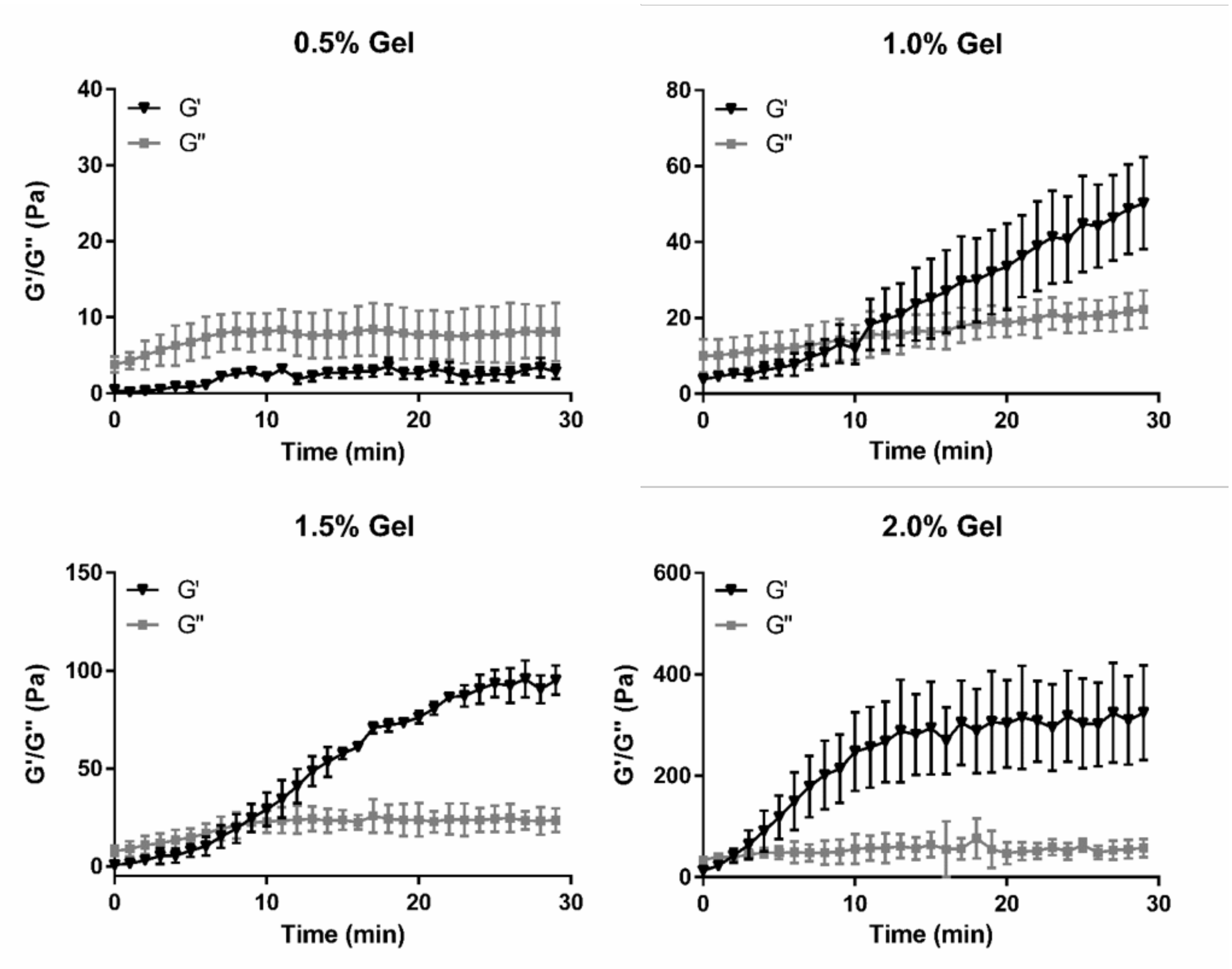

Figure S2. Rheological analyses (G′ and G″) of bulk HA hydrogels made of various concentrations (0.5, 1.0, 1.5, and 2.0 w/v %) of HyStem® precursors. (The results represent means ± SEM. N ≥ 3.)

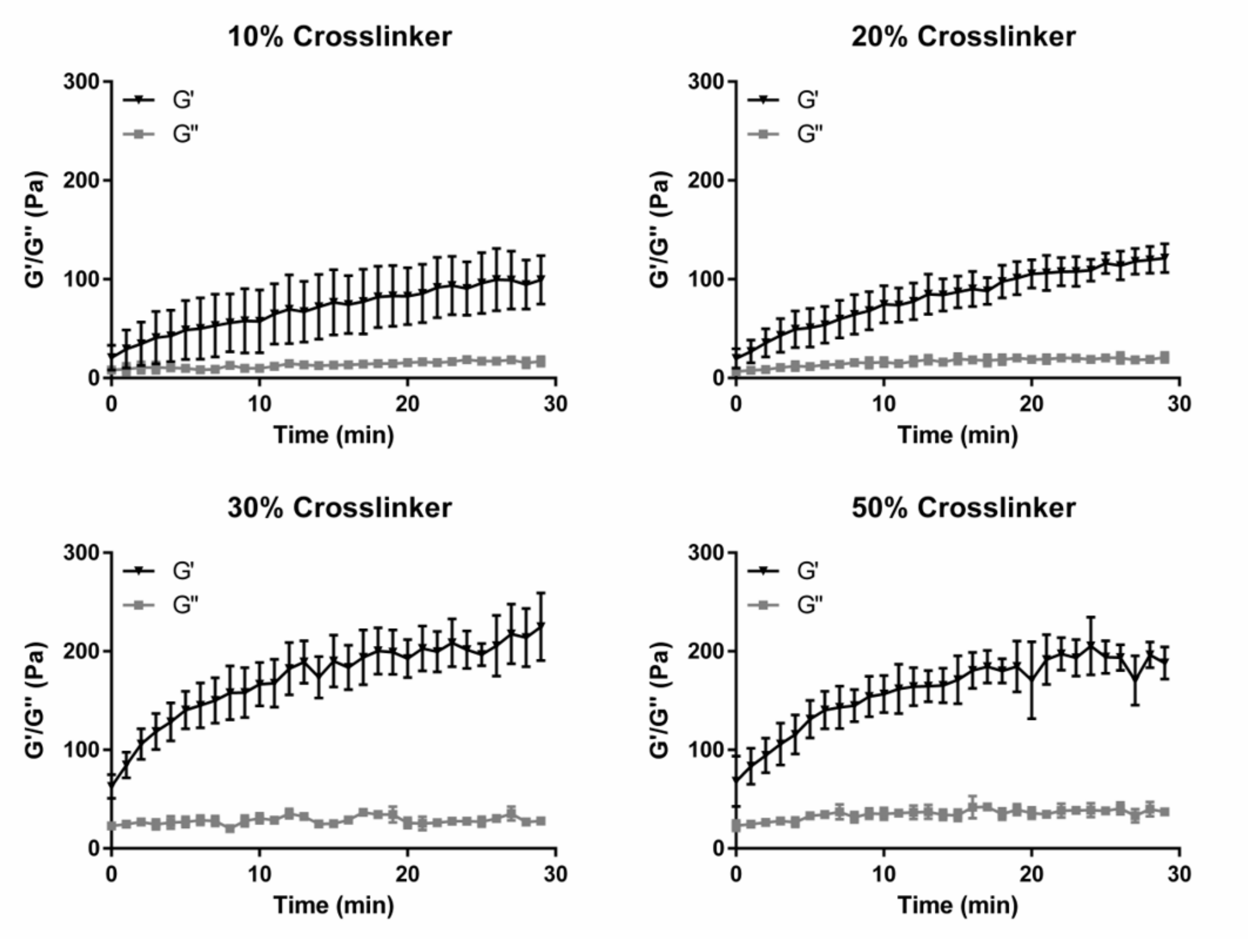

Figure S3. Rheological analyses (G′ and G″) of granular HA hydrogel composites made of 2.0 w/v % hydrogel granules and secondary crosslinkers at 10, 20, 30, or 50 w/v % (relative to the primary crosslinker concentration). (The results represent means ± SEM. N = 3.)

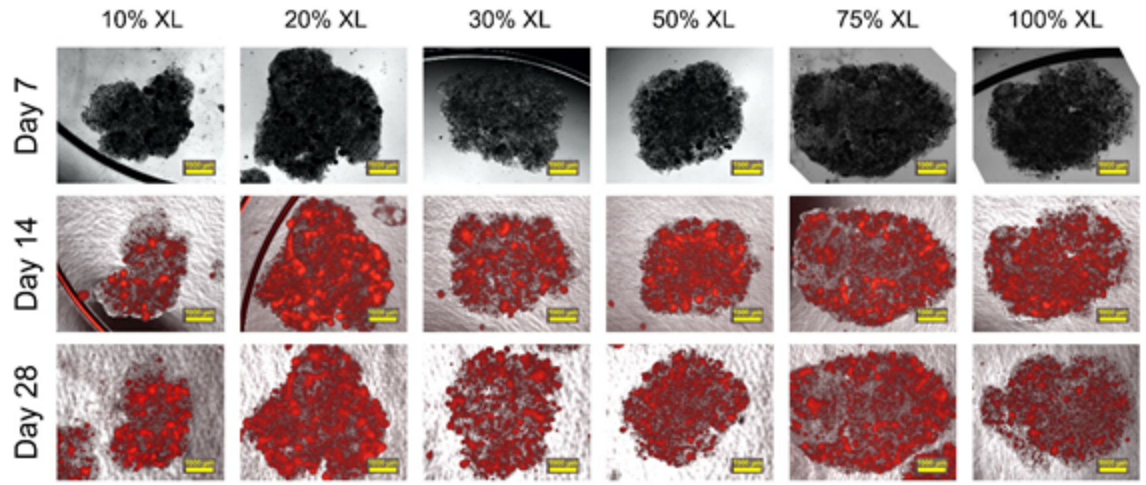

Figure S4. Cell encapsulation in granular hydrogel composites. SH-SY5Y cells expressing mCherry fluorescent protein were encapsulated in granular hydrogel composites made with varying amounts of secondary crosslinker (10, 20, 30, 50, 75, and 100 w/v % of secondary crosslinkers) and were imaged at Day 7, 14 and 28 time points (scale bars = 1 mm) (XL: crosslinker).

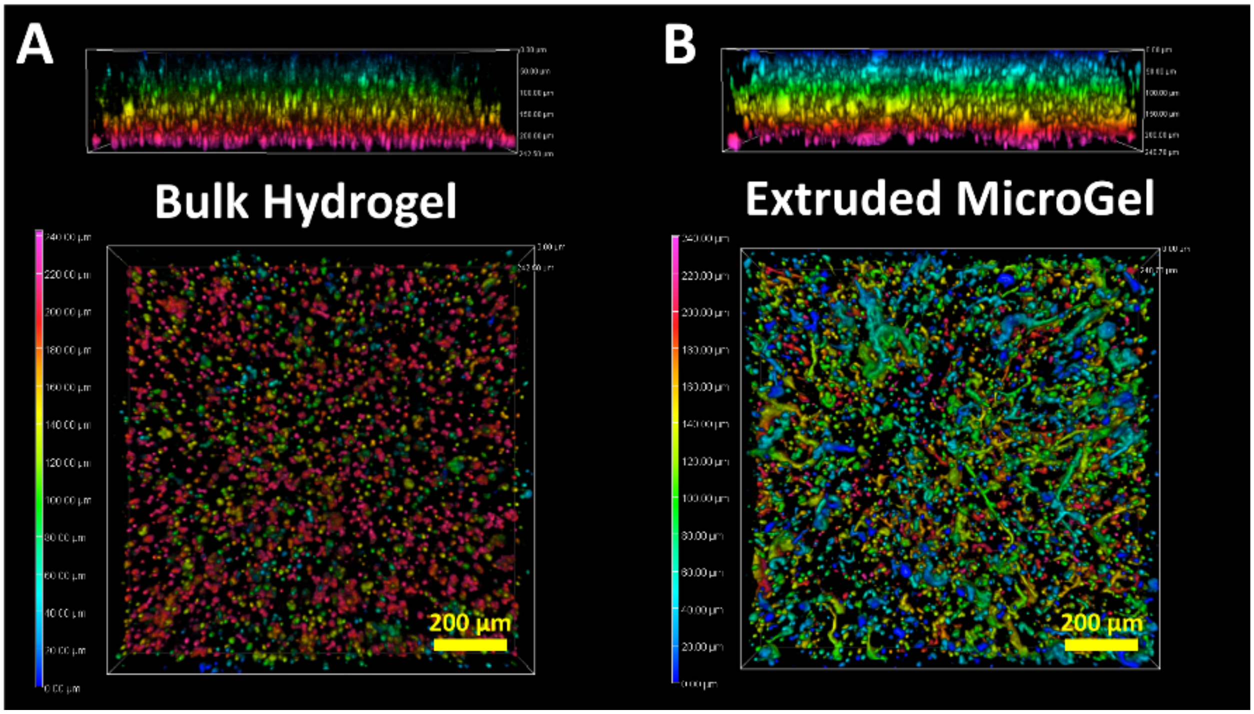

Figure S5. SH-SY5Y cells (10^7 cells/ml) mixed into bulk and granular gel discs (240 μm thickness) can be spaced homogenously during seeding (scale bars = 200 μm). The top panels represent XZ projection images of the Z stack and the color represents the z-depth of the gel stacks. From the XZ projection image and the color map, it was shown that cells in granular gel discs were distributed homogenously in all three dimensions.

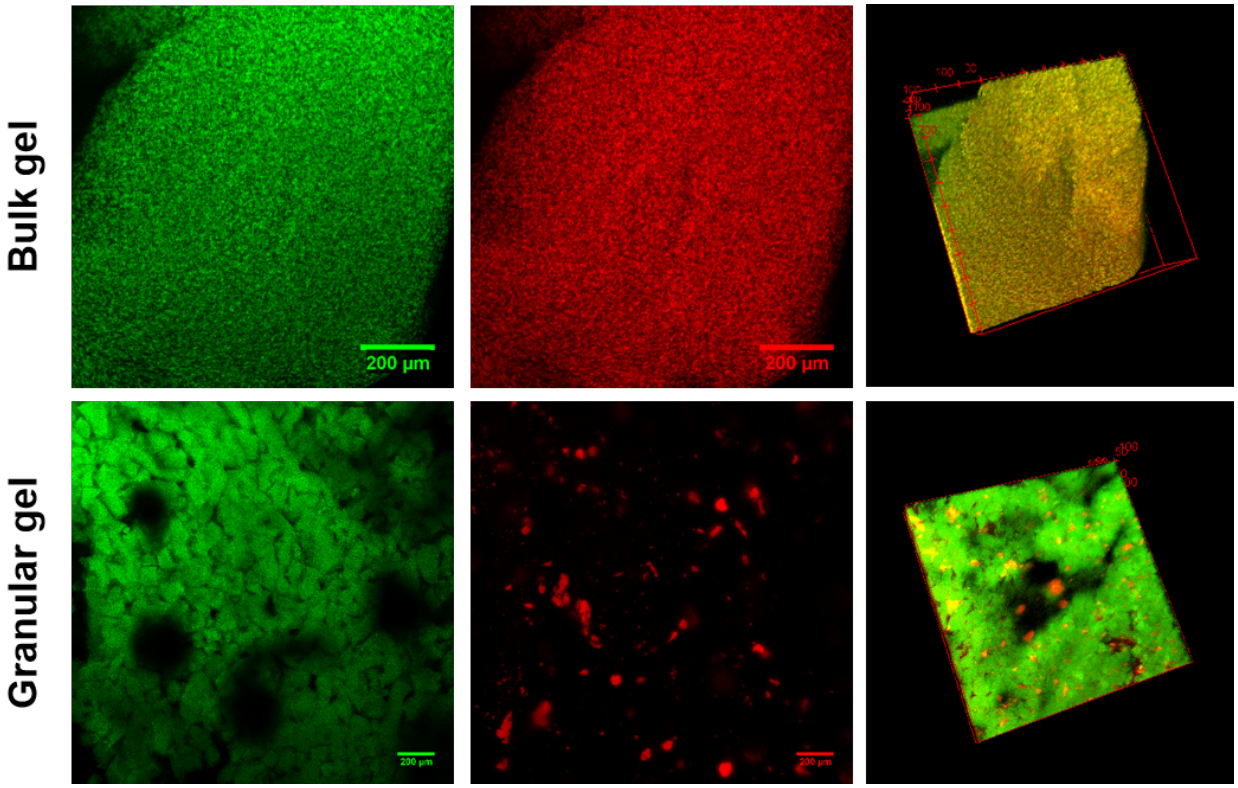

Figure S6. The internal structure of bulk and granular hydrogels was characterized by confocal microscopy with incorporation of fluorescent beads at different stages of crosslinking. For bulk hydrogels, both green and red fluorescent beads were incorporated into the hydrogel in 1:1 ratio during hydrogel fabrication. For granular hydrogels, green fluorescent beads were incorporated into the hydrogel granule during primary crosslinking and the same amount of red fluorescent beads were incorporated into the granular hydrogel composite during secondary crosslinking. While the fluorescent beads were homogenously distributed in the bulk hydrogel, the granular

hydrogel provides interconnected void spaces (indicated by red fluorescent beads) in the scaffold (scale bars = 200 μm). Readers are also referred to previous studies, which reported internal morphologies of bulk HA hydrogels using scanning electron microscopy (SEM) [1, 2]. However, the techniques used for SEM preparation (dehydration and freezing, etc.) can change the hydrogel structure and may not reflect the original hydrogel state [3].

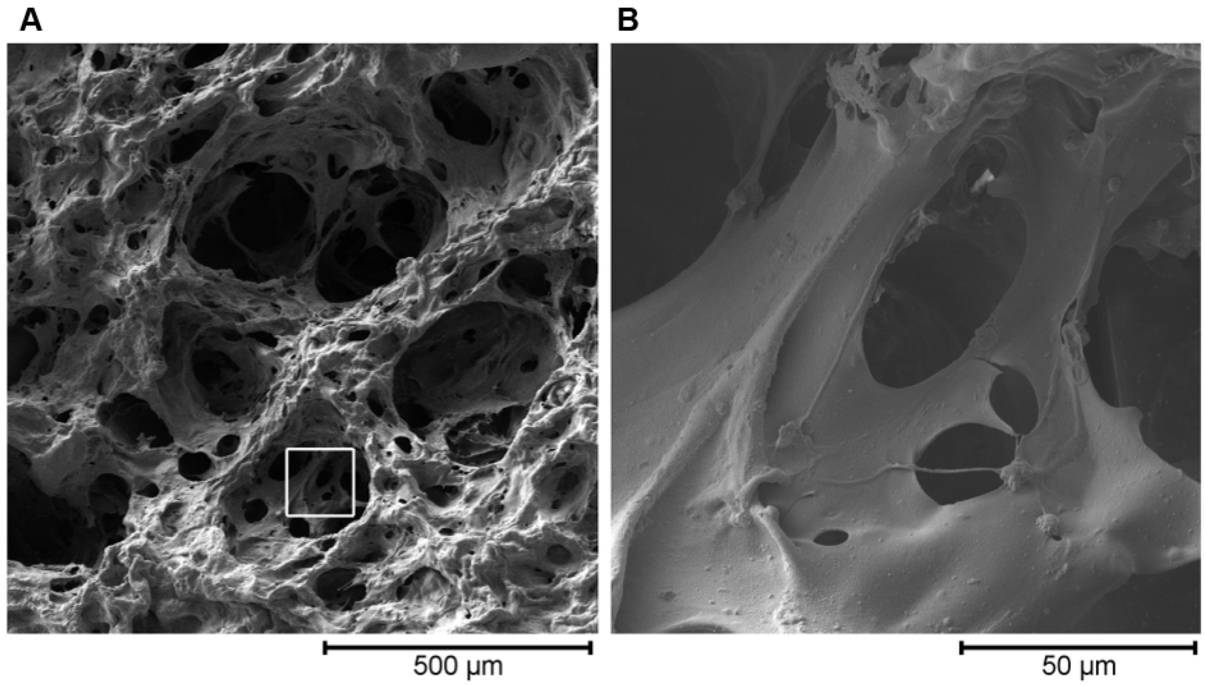

Figure S7. Granular architecture provides interconnected micropore or mesopore systems, which may provide a more permissive environment for cell migration. (A) SEM image of the micropore/mesopore system in the granular hydrogel composite. (B) A zoomed-in field in (A), marked using a white square, revealing neurites crossing over pores.

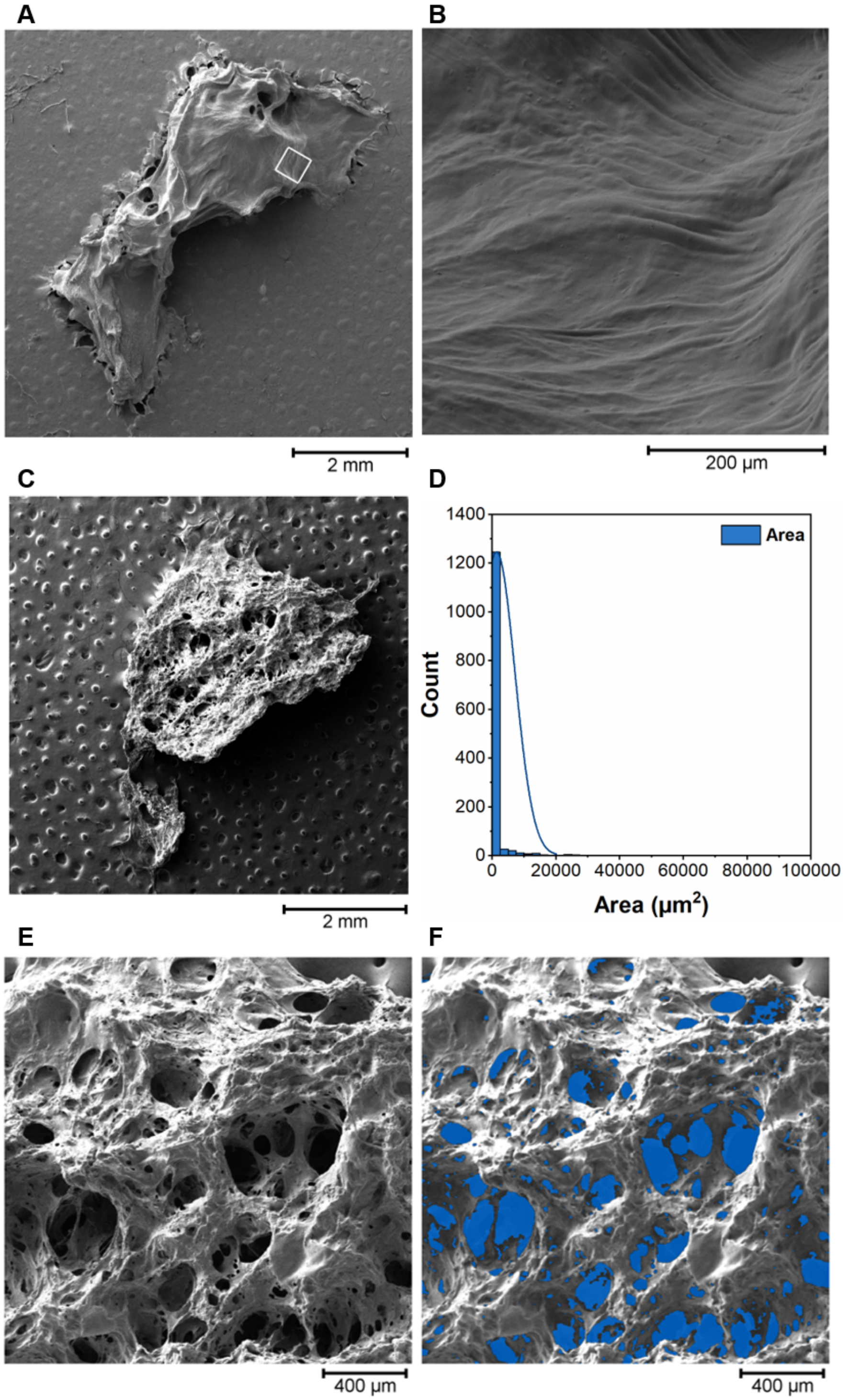

Figure S8. Characterization of bulk and granular hydrogel composites using scanning electron microscopy (SEM). SEM images of (A) a bulk hydrogel and (B) a zoomed-in field selected in (A), marked using a white square. (C) SEM imaging of granular hydrogel composites showed

microporous and interconnected structure compared to the bulk hydrogel. The distribution of the surface area of the pores was shown in (D) and the images were analyzed using 3D imaging software Avizo version 2020.1. (E) The secondary electron SEM images were filtered using Median Filter, segmented using Watershed Segmentation and the Segmentation Editor was used to remove any background signal. (F) Label Analysis module was used to extract the surface area of segmented pores (marked in blue) based on pixel intensity (n=3, 1353 pores were analyzed).

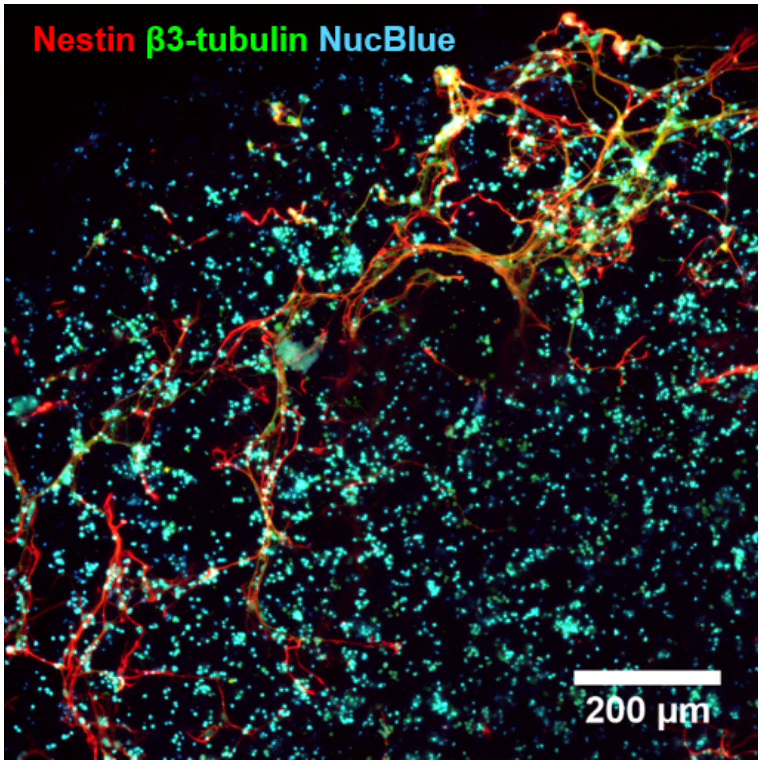

Figure S9. Cell encapsulation of hiPSC (line 010S-1)-derived NPCs in granular hydrogel composites for 1 month (scale bars = 200 μm; Nestin, red; Tuj1, Green; NucBlue, Blue).

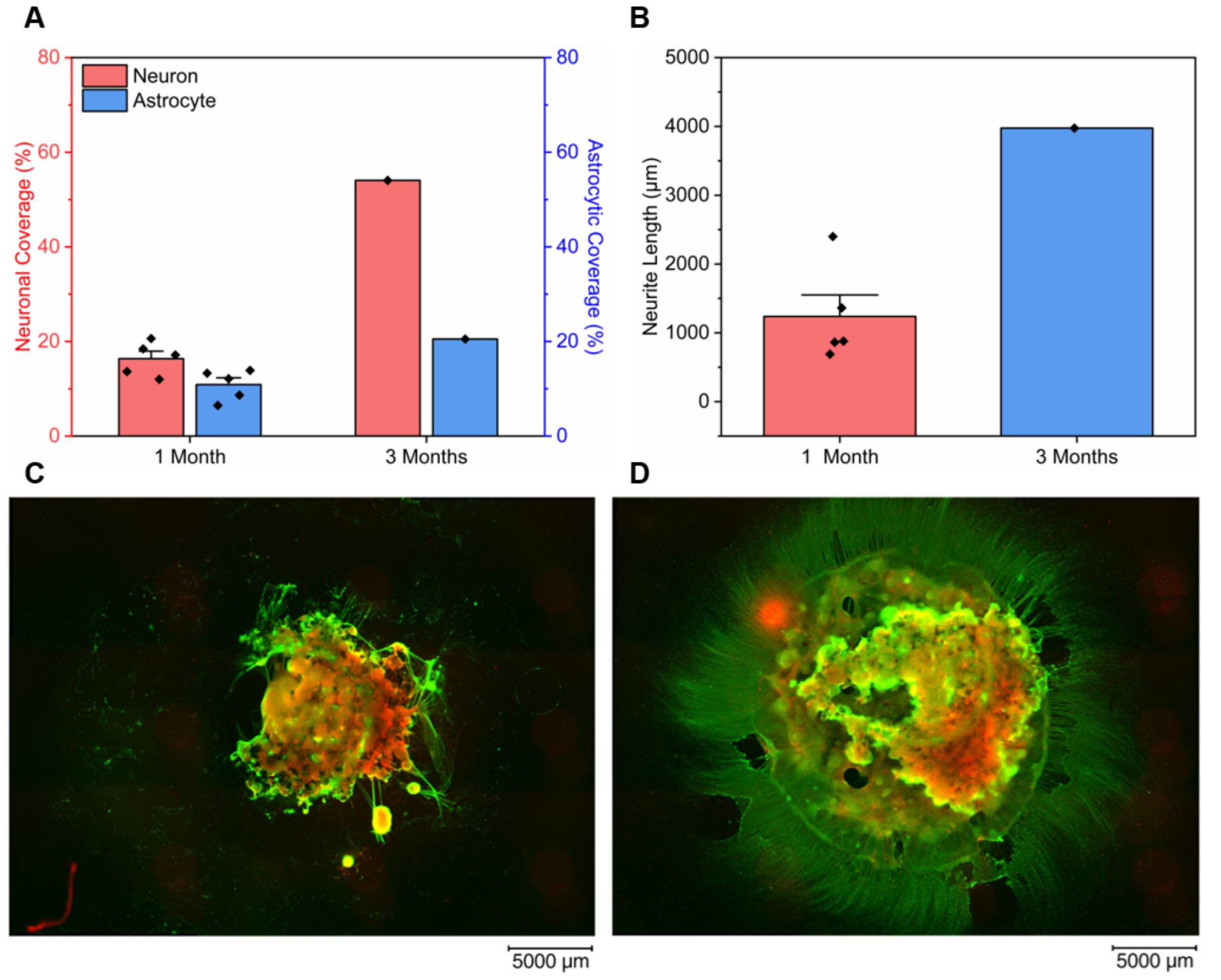

Figure S10. Neural differentiation and neurite extension in longer term culture of granular hydrogel composites. (A) Neuronal and astrocytic coverage in granular hydrogel composites quantified by surface coverage area per field (%) analyzed by Image J after 1 month and 3 months culture (n=5 for 1 month culture and n=1 for 3 months culture). (B) Neurite/neurite bundle extension as indication for neuronal maturation after 1 month and 3 months culture was quantified by Filament Editor workroom of Avizo (10 neurites/neurite bundles were analyzed per sample; n=5 for 1 month culture and n=1 for 3 months culture). Epifluorescent images of granular microgel neural culture after (C) 1 month and (D) 3 months culture (scale bar = 5000 μm). Immunostaining reveals neurons (Tuj1, Green) and astrocytes (GFAP, Red).

Materials and Methods

Table S1. Equivalent cell seeding densities for 2D controls and 3D cubic hydrogel models.

2D ***(A^2 cells/cm2)***	***3D*** ***(A^3 cells/cm3)***
10,000	1,000,000
20,000	2,828,427
30,000	5,196,152
40,000	8,000,000
50,000	11,180,340
100,000	31,622,777

Table S2. List of antibodies used for immunostaining.

Antibody	***Host***	***Catalogue Number***	***Company***	***Dilution***
Nestin	mouse IgG-1	MAB5326	Millipore	1:500
GFAP	rabbit (poly)	G9269	Sigma-Aldrich	1:1000
Neurofilament	chicken (poly)	ab4680	Abcam	1:1000
βIII-Tubulin	mouse IgG-2b	T5076	Sigma-Aldrich	1:1000

SH-SY5Y Neuron Culture

The SH-SY5Y, a human neuroblastoma cell line, was obtained from the European Collection of Cell Cultures (cat. no: 94030304) and the generation of mCherry expressing SH-SY5Y cells and the cell culture protocol are described in a previous publication [4]. The cells were cultured in complete media composed of RPMI 1640 with glutamine (GE Healthcare, UK), supplemented with 0.1 mM nonessential amino acids (GE Healthcare), 10 v/v % foetal bovine serum (FBS; Gibco, UK), and 1 v/v % penicillin (100 U/ml) with streptomycin (100 mg/ml) (GE Healthcare). The culture medium was exchanged every 2–3 d and cultures were passaged when they reached 80% confluency. Cells were differentiated in T75 flasks (Corning, USA) for 7 d through exposure

to 10 μM all-trans-retinoic acid (RA; Sigma-Aldrich, UK) and harvested using 1x Trypsin (0.5 w/v %) with EDTA (0.02 w/v %) (GE Healthcare) for seeding.

Scanning Electron Microscopy (SEM) Sample Preparation and SEM Imaging

Each sample was fixed in 4.0 v/v % paraformaldehyde (Sigma-Aldrich) in PBS for 30 min at room temperature and rinsed three times with PBS to remove residual paraformaldehyde. The hydrogels were then mounted on aluminum SEM stubs using carbon tape. Samples were coated in a vacuum chamber with gold (Au) and palladium (Pd) with a thickness of around 20 nm using SC7620 sputter coater (Quorum Technologies, U.K.) and analyzed using a SEM Tescan Lyra 3 (Tescan, Czech Republic) using a voltage of 5 keV.

SI References

[1] A. Skardal, S.V. Murphy, K. Crowell, D. Mack, A. Atala, S. Soker, A tunable hydrogel system for long-term release of cell-secreted cytokines and bioprinted in situ wound cell delivery, Journal of Biomedical Materials Research Part B: Applied Biomaterials 105(7) (2017) 1986-2000.

[2] N.C. Hunt, D. Hallam, A. Karimi, C.B. Mellough, J. Chen, D.H.W. Steel, M. Lako, 3D culture of human pluripotent stem cells in RGD-alginate hydrogel improves retinal tissue development, Acta Biomaterialia 49 (2017) 329-343.

[3] Z. Kaberova, E. Karpushkin, M. Nevoralová, M. Vetrík, M. Šlouf, M. Dušková-Smrčková, Microscopic Structure of Swollen Hydrogels by Scanning Electron and Light Microscopies: Artifacts and Reality, Polymers 12(3) (2020).

[4] J.H. George, D. Nagel, S. Waller, E. Hill, H.R. Parri, M.D. Coleman, Z. Cui, H. Ye, A closer look at neuron interaction with track-etched microporous membranes, Scientific Reports 8(1) (2018) 15552.
